# The Tumor Suppressor *MTUS1*/ATIP1 Modulates Tumor Promotion in Glioma: Association with Epigenetics and DNA Repair

**DOI:** 10.3390/cancers13061245

**Published:** 2021-03-12

**Authors:** Nikhil Ranjan, Vimal Pandey, Manas Kumar Panigrahi, Lukas Klumpp, Ulrike Naumann, Phanithi Prakash Babu

**Affiliations:** 1 Laboratory of Neuroscience, Department of Biotechnology & Bioinformatics, School of Life Sciences, University of Hyderabad, Telangana 500046, India; nranjan03@uohyd.ac.in (N.R.); vimal_2808@yahoo.com (V.P.); 2 Laboratory of Molecular Neuro-Oncology, Department of General Neurology, Hertie-Institute for Clinical Brain Research and Center Neurology, University of Tuebingen, Otfried-Mueller-Str. 27, 72076 Tuebingen, Germany; 3 Department of Neurosurgery and Pathology, Krishna Institute of Medical Sciences (KIMS), Secunderabad, Telangana 500003, India; manasp1966@gmail.com; 4 Department of Radiation Oncology, University Hospital Tübingen, 72076 Tübingen, Germany; lukas.klumpp@outlook.com

**Keywords:** glioma, recurrence, *MTUS1*/ATIP1, hypermethylation, decitabine, cell motility, DNA repair

## Abstract

**Simple Summary:**

Despite multidisciplinary treatments, survival remains poor in glioma patients. Although novel therapeutic approaches are being explored, no outstanding effects on the survival have been achieved so far, which substantiates the need to develop new therapeutic strategies. To understand the mechanisms responsible for its high malignancy and obligatory recurrence, we examined the impact of *MTUS1*, a tumor-suppressor gene (TSG), coding for ATIP1, in glioma malignancy as well as how its expression might influence glioma therapy. We confirmed that in glioma cells, elevated ATIP1 expression damps tumor progression by mitigating proliferation and motility. Additionally, *MTUS1*/ATIP1 can be used as a biological marker to predict therapy outcomes. In glioma cell lines, glioma sphere cultures (GSC), high-grade glioma (HGG) and especially in glioma recurrence, *MTUS1*/ATIP1 expression is downregulated, probably by promoter hypermethylation. However, in GBM, high ATIP1 expression might interfere with radiation-therapy since elevated expression of *MTUS1*/ATIP1 drives double-strand break (DSB) DNA repair.

**Abstract:**

Glioblastoma (GBM) is a highly aggressive brain tumor. Resistance mechanisms in GBM present an array of challenges to understand its biology and to develop novel therapeutic strategies. We investigated the role of a TSG, *MTUS1*/ATIP1 in glioma. Glioma specimen, cells and low passage GBM sphere cultures (GSC) were analyzed for *MTUS1*/ATIP1 expression at the RNA and protein level. Methylation analyses were done by bisulfite sequencing (BSS). The consequence of chemotherapy and irradiation on ATIP1 expression and the influence of different cellular ATIP1 levels on survival was examined in vitro and in vivo. *MTUS1*/ATIP1 was downregulated in high-grade glioma (HGG), GSC and GBM cells and hypermethylation at the ATIP1 promoter region seems to be at least partially responsible for this downregulation. ATIP1 overexpression significantly reduced glioma progression by mitigating cell motility, proliferation and facilitate cell death. In glioma-bearing mice, elevated *MTUS1*/ATIP1 expression prolonged their survival. Chemotherapy, as well as irradiation, recovered ATIP1 expression both in vitro and in vivo. Surprisingly, ATIP1 overexpression increased irradiation-induced DNA-damage repair, resulting in radio-resistance. Our findings indicate that *MTUS1*/ATIP1 serves as TSG-regulating gliomagenesis, progression and therapy resistance. In HGG, higher *MTUS1*/ATIP1 expression might interfere with tumor irradiation therapy.

## 1. Introduction

Glioblastoma (GBM) are highly malignant and therapy-resistant primary brain tumors of adults with an unfavorable prognosis and a median survival of less than 20 months, even at best care and optimal therapy [1]. The malignancy of GBM is based on its characteristics. GBM are highly immunosuppressive and massively proliferating tumors that grow invasively into the surrounding brain parenchyma. Additionally, recurrent gliomas are mainly resistant towards irradiation and most chemotherapeutics, based on the existence of an extremely resistant subpopulation of GBM cells known as glioma stem cells, which are responsible for tumor initiation and recurrence [2,3]. The multistage process of tumorigenesis and tumor progression includes the progressive acquisition of genetic and epigenetic alterations, resulting in the activation of proto-oncogenes and inactivation of tumor suppressor genes (TSG). In tumor cells, genomic alterations include deletions, amplifications and mutations [4]. At the epigenetic level, besides histone-(de)acetylation, DNA-methylation plays an essential role in the regulation of gene expression. In glioma, alterations in DNA-methylation include hypomethylation of oncogenes and hypermethylation of TSG, which might interplay with the activation of cancer-associated signaling pathways [5,6,7,8]. Characteristics of glioma are the activation of the phosphatidylinositol 3-kinase (PI3K)/AKT, mitogen-activated protein kinase (MAPK) and epidermal growth factor receptor (EGFR) signaling pathways which regulate proliferation, survival and cancer cell motility, and which are interconnected to several other cancer-associated processes such as autophagy, ROS production, DNA damage and repair, chromosomal instability and metastasis [9,10,11,12]. Supplementary to surgical resection of the tumor, the standard treatment of GBM includes irradiation and chemotherapy. Whilst irradiation induces single and double-strand DNA breaks, temozolomide (TMZ), the standard therapeutic drug to treat glioma, methylates guanine residues leading to activation of DNA repair mechanisms. Repair mechanisms that lead to resistance and that are common in glioma cells include DNA mismatch repair, homologous recombination (HR), non-homologous end joining (NHEJ) as well as the removal of guanine methylation by O^6^-methylguanine-DNA methyltransferase (MGMT) [13].

Loss of function mutations of TSG drive cells towards tumor progression [14,15]. The expression of the microtubule-associated tumor suppressor (*MTUS1*) gene has been reported to be lost in various cancer types like colon, ovarian, pancreas, bladder, gastric and lung cancer [16]. *MTUS1* encodes a family of angiotensin II (AT2) receptor-interacting proteins (ATIP). Alternative splicing results in five different isoforms of ATIP (ATIP1, ATIP2, ATIP3a, ATIP3b, and ATIP4) [17,18,19]. Besides the important role of maintaining vascular integrity and homeostasis at the level of the angiotensin II (AngII)/angiotensin receptor system (AT_x_R) through the Renin Angiotensin Aldosterone System (RAAS) [20], RAAS also plays a crucial role in cancer biology and affects tumor growth by remodeling the tumor microenvironment [21]. In oral squamous cell carcinoma, ATIP1 is regulated by p53 and inhibits epidermal growth factor (EGF)-mediated ERK phosphorylation, cell proliferation and migration, indicating that ATIP1 serves as a tumor suppressor [22,23]. In the central nervous system (CNS), ATIP1 is the most abundant transcript expressed in all brain regions except the cerebellum and fetal brain and is involved in neural differentiation [18,24,25], suggesting that it might play a crucial role in physiological functions via different intracellular mechanisms. Recent studies have shown that after angiotensin II receptor (AT_2_R) stimulation, ATIP1 interacts with the Src homology region 2 domain-containing phosphatase-1 (SHP-1). This complex is translocated into the nucleus where it transactivates the ubiquitin-conjugating enzyme methyl methanesulfonate sensitive 2 (*MMS2/UBE2V2*), involved in the error-free-post-replication repair pathway [26].

In the present study, we demonstrate that *MTUS1*/ATIP1 expression is reduced or lost in high-grade glioma (HGG) tissue and patient-derived low passage glioma sphere cultures (GSC). Elevated expression of ATIP1 in glioma cells extenuates tumor-driving molecular signaling pathways and mitigates proliferation as well as migration, invasion and clonogenic survival. Bisulfite sequencing (BSS) of the 5′ upstream *MTUS1*-transcription region indicates the plausible influence of DNA-methylation in *MTUS1*/ATIP1 expression. We ascertained that glioma therapy induces ATIP1 expression. For this, we analyzed its role in the repair of irradiation-induced DNA damage and its association to p53. Our findings support that in GBM, *MTUS1*/ATIP1 acts as a TSG, and we believe that a detailed elucidation of the role of *MTUS1*/ATIP1 in GBM might help to develop new strategies that target *MTUS1*/ATIP1-associated pathways and that might help to slow down or even block the progression of glioma.

## 2. Results

### 2.1. Assessment of MTUS1/ATIP1-Expression in Glioma

**Figure 1:**
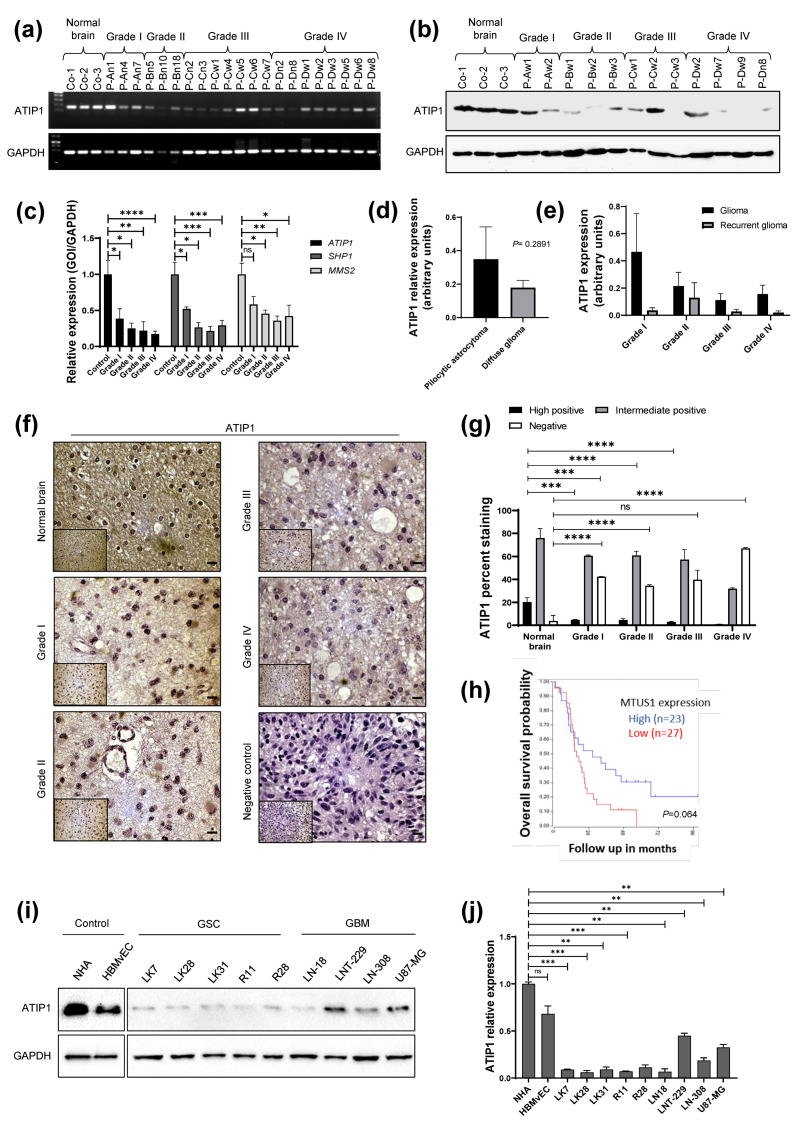
ATIP1 expression in normal human brain and glioma. (**a**,**b**). *ATIP1* PCR (**a**) and Western blot (**b**) in glioma specimens and normal brains. (**c**). q-RT-PCR of *ATIP1* (left), *SHP1* (middle) and *MMS2* (right) in the normal human brain (control; n = 5) or glioma specimen of different WHO grades (grade I, n = 6; grade II, n = 12; grade III, n = 15; grade IV, n = 15) (n = 3, SEM; ns: not significant; * *p* < 0.05, ** *p* < 0.01, *** *p* < 0.001, **** *p* < 0.0001). (**d**). Immunoblot-based quantification of ATIP1 expression in pilocytic glioma (n = 6) and diffuse glioma (grades II-IV, n = 70). (**e**). ATIP1 protein expression in the tissue of original and recurrent glioma samples of different WHO grades as determined by immunoblot. (**f**). Immuno-histopathological analysis of ATIP1 expression in normal human brain and glioma of different WHO grades. Representative pictures are shown. (**g**). Quantification of immuno-histopathologically determined ATIP1 expression analyses in glioma and normal brain tissue as percentages of staining (bar = 100 μm; normal brain, n = 4; WHO grade I, n = 4; grade II, n = 12; grade III, n = 18; grade IV, n = 15; ns: not significant; * *p* < 0.05, ** *p* < 0.01, *** *p* < 0.001, **** *p* < 0.0001). (**h**). Overall survival probability of glioma patients determined by R2 database mining of glioma samples. Higher *MTUS1* expression (n = 23) results in better survival compared to patients presenting low *MTUS1* expression (n = 27). (**i**). ATIP1 protein expression in low passage GSC, glioma cells (GBM) and non-neoplastic control cells of brain origin (NHA: Normal Human Astrocytes, HBMvEC: Human Brain Microvascular Endothelial Cells; n = 3, one representative experiment is shown). (**j**). Quantification of ATIP1 protein expression in GBM, GSC and control cells (n = 3, SEM; ns: not significant, ** *p* < 0.01, *** *p* < 0.001).

ATIP1 mRNA was significantly downregulated in human glioma specimens, this correlating well with reduced levels of the protein. Additionally, ATIP1 expression, as well as that of the ATIP1 upstream (*SHP-1*) and downstream regulator (*MMS2/UBE2V2*) proteins, correlated with glioma progression. In HGG these genes were downregulated approximately threefold compared to normal brain tissue (Figure 1a–c, Figure S1a, Table S6). Although not significant, we observed a trend towards lower ATIP1 expression in diffuse glioma grades specimen (grade II-IV, n = 70) as compared to the pilocytic astrocytoma specimens (grade I, n = 6) (Figure 1d). ATIP1 expression was also reduced in recurrent glioma of all WHO grades when compared to the original tumors (Figure 1e). In glioma specimens of different WHO grades, ATIP1 was downregulated in a grade-dependent manner (Figure 1f,g) indicating that downregulation of ATIP1 is correlated with an elevated malignancy of glioma. In order to analyze the effects of IDH mutations on ATIP1 expression, we performed a correlation analysis and found that ATIP1 was downregulated in both IDH^WT^ and IDH^mut^ HGG compared to low-grade glioma (LGG) of the same IDH status (Figure S1b, Table S1). To determine whether elevated ATIP1-expression correlates to better survival we used the R2-database. With a p-value of 0.064, lower ATIP1 mRNA levels in human glioma correlate with a worse outcome (Figure 1h). We also determined whether lower ATIP1 expression in our cohort of glioma is associated with worse survival. Even being not significant due to the small number of cases in the ATIP1^high^ cohort, ATIP1^high^ glioma patients showed a slight trend towards a prolongated median survival (median survival not reached so far) compared to ATIP1^intermediate/low^ patients (48 months) (Figure S2a). Using the R2 database as well as the Indian cohort of glioma patients, we also determined whether in glioma patients there are age- or gender-specific differences in *MTUS1*/ATIP1 expression. We detected no significant gender or age-related changes in *MTUS1*/ATIP1 expression. However, putatively due to the low number of patients in each group, no significant correlation of gender- or age-specific ATIP1 expression with patient’s survival was observed. Interestingly, in the group of elderly (>65 years) that harbor mainly HGG, no ATIP1^high^ patient was present (Figure S2b–e).

In a panel of patient-derived low passage GSC, established GBM cells and primary non-neoplastic human brain cells, ATIP1 was expressed in non-neoplastic but was downregulated in all tumor cells (Figure 1i,j). We also correlated MGMT promoter methylation data from our GSC and glioma cell lines with ATIP1 expression. Interestingly, cells harboring a methylated MGMT promoter showed higher ATIP1 expression compared to cells presenting an unmethylated MGMT promoter (Figure S1c, Table S2).

### 2.2. Methylation Status of the 5′ upstream MTUS1-Transcription Start Site

**Figure 2:**
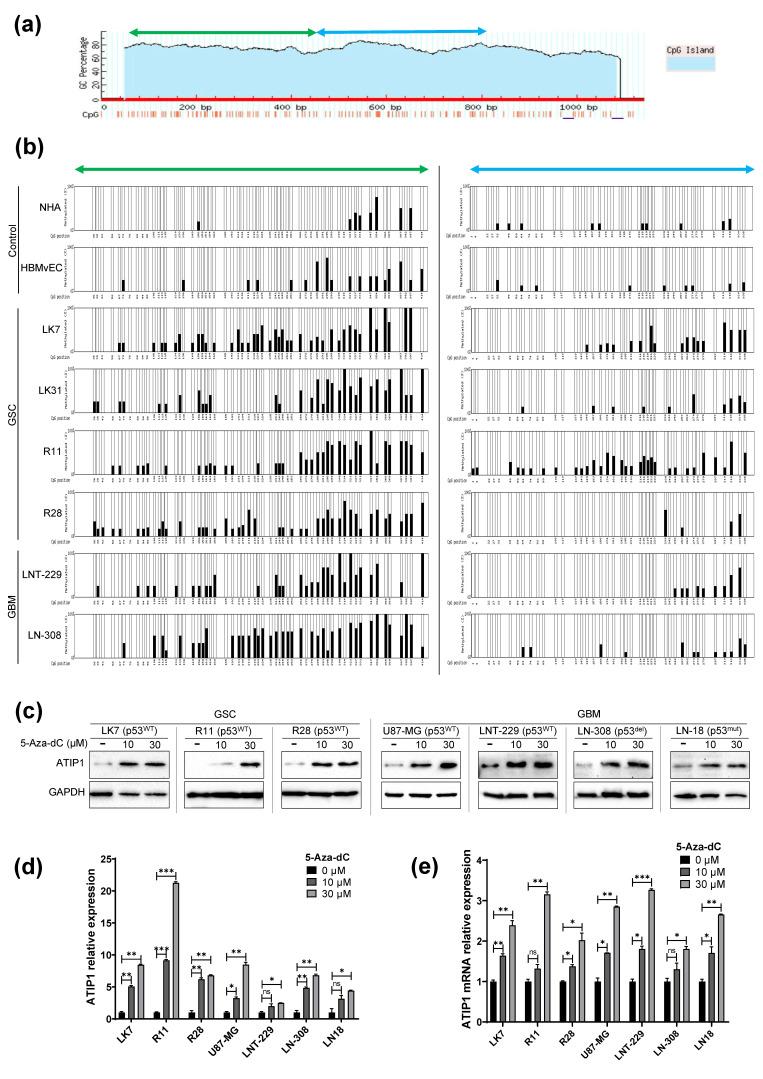
In glioma cells, the *MTUS1* promoter region shows hypermethylation. (**a**) Localization of a predicted CpG island in the 5′ upstream *MTUS1*-TSS region (red). The green and blue labelled regions were analyzed for BSS and indicate the proximal (blue, 57 CpG) and distal (green, 41 CpG) part of the CpG island. (**b**) Percentage of methylation in non-neoplastic primary brain cells (control), low passage GSC and glioma cells. (**c**–**e**) Decitabine (5-Aza-dC) treatment leads to the re-expression of ATIP1 both on mRNA and protein levels in GBM and GSC. (**c**) Immunoblot of ATIP1 in GSC and GBM cells after treatment with decitabine as described in the material and methods part. GAPDH serves as the loading control (n = 3, one representative experiment is shown). (**d**) Quantification of ATIP1 protein upon decitabine treatment at increasing concentrations (n = 3, SEM; ns: not significant, * *p* < 0.05, ** *p* < 0.01, *** *p* < 0.001). (**e**) Relative expression of ATIP1 mRNA expression post decitabine treatment (n = 3, SEM; ns, * *p* < 0.05, ** *p* < 0.01, *** *p* < 0.001).

Since the downregulation of TSG in cancer cells is often provoked by hypermethylation [27], we examined whether the downregulation of ATIP1 expression in glioma might be induced by methylation of its promoter region. Using MethPrimer we identified an approximately 1000 bp putative CpG island 5′ upstream of the *MTUS1* transcription start site (TSS) and determined the methylation status of 57 CpG in the proximal and 41 CpG in the distal part (Figure 2a). Methylation pattern analyses revealed hypomethylation of this region in non-neoplastic cells whilst GSC and GBM cells showed hypermethylation, making the methylation pattern in the proximal part more variable (Figure 2b). This indicates that the *MTUS1* downregulation we observed might be a result of hypermethylation in the 5′ upstream *MTUS1*-TSS region. To determine whether a lower ATIP1 expression is dependent on this hypermethylation, we subsequently treated the cells with decitabine (5-Aza-dC), a hypomethylating agent, which resulted in the re-expression of ATIP1 at the transcriptional and translational level in all GBM and GSC cells tested so far, irrespective of the p53 status of these cells (Figure 2c–e), suggesting that hypermethylation is partially responsible for *MTUS1*/ATIP1 downregulation in HGG.

### 2.3. ATIP1 Modulates Cell Proliferation, Motility and Survival

**Figure 3:**
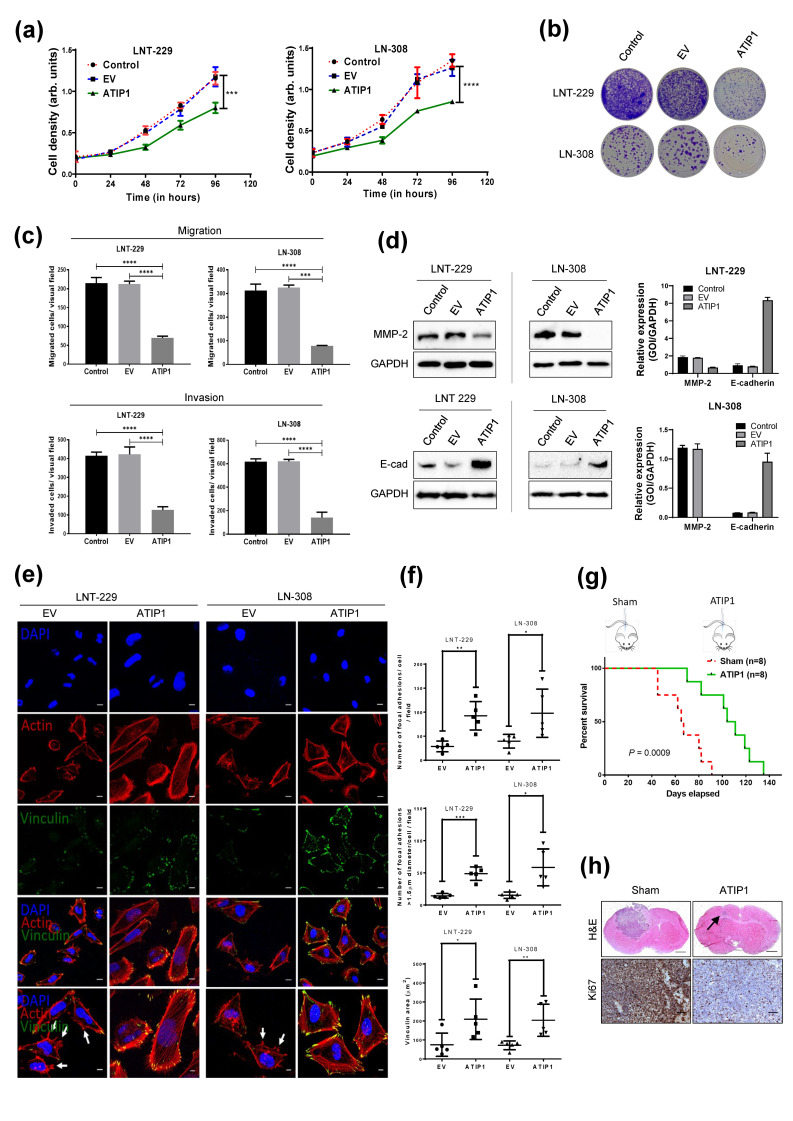
ATIP1 overexpression modulates several pro-tumorigenic characteristics. Parental (control), pcDNA3.1 (EV) or pcDNA3.1-ATIP1 (ATIP1) stably transfected LNT-229 and LN-308 cells were subjected for functional assays. (**a**) Cell density was determined by crystal violet staining at the indicated time points (n = 3, SEM, *** *p* < 0.001, **** *p* < 0.0001). (**b**) Clonogenic survival (n = 3, one representative experiment is shown). (**c**) Quantification of transwell cell migration and invasion (n = 4; SEM, *** *p* < 0.001, **** *p* < 0.0001). (**d**) Western blot of MMP-2 (upper panel) and E-cadherin (lower panel), GAPDH serves as a loading control (n = 3, one representative experiment is shown). The right panels respectively show the quantification of MMP-2 and E-Cadherin (n = 3, SEM). (**e**) Immunofluorescence analyses of actin fibers (red) and vinculin (green) indicate the higher amount of focal adhesion complexes in ATIP1 overexpressing LNT-229 and LN-308 pcDNA3.1 (EV) and pcDNA3.1-ATIP1 stably transfected cells (scale = 100 µm). Arrows indicate lamellipodia and filopodia. (**f**). Quantification of focal adhesion complexes (upper panel), large focal adhesion contact points (middle panel) and of the total area stained for vinculin (lower panel; n = 6; * *p* < 0.05, ** *p* < 0.01, *** *p* < 0.001). (**g**) Survival of mice harboring either pcDNA3.1 (sham) or pcDNA3.1-ATIP1 stably transfected (ATIP1) LNT-229 gliomas (n = 8 mice per group). (**h**) Elevated ATIP1 expression reduces tumor growth and proliferation of tumor cells. H&E (upper panel, scale = 1000 µm) and Ki67 (lower panel, scale = 500 µm) staining of mice harboring gliomas developed after intrastriatal implantation of pcDNA3.1 (EV) or pcDNA3.1-ATIP1 stably transfected LNT-229 cells (n = 3 mice per group, one representative picture is shown). The arrow indicates the tumor region in an LNT-229-pcDNA3.1-ATIP1-glioma bearing mouse.

To elucidate the biological role of ATIP1, we generated ATIP1 overexpressing GBM cells (Figure S3) and analyzed cell growth, clonogenic survival, migration and invasion in parental, pcDNA3.1 and pcDNA3.1-ATIP1 stably transfected GBM cells. As indicated in Figure 3a, ATIP1 overexpressing LNT-229 and LN-308 cells grew significantly slower than parental or control cells and showed a reduced capability of forming colonies (Figure 3b). Reduced growth was accompanied by an elevated population of cells arrested in the G2/M cell cycle phase (Figure S4a). Additionally, ATIP1-overexpression significantly mitigated the capabilities of the cells to migrate and invade (Figure 3c, Figures S4b,c and S5), accompanied by the downregulation of matrix-metalloproteinase-2 (MMP-2) and upregulation of E-cadherin (Figure 3d). Reduced motility in ATIP1 overexpressing cells was paralleled by a significant upregulation of vinculin positive large focal adhesions complexes (LFAC) which was associated with actin stress fibers and by a lower number of filopodia and lamellipodia (Figure 3e,f), indicating a tighter adhesion of ATIP1 overexpressing cells compared to control cells.

To further evaluate the tumor suppressor activity of ATIP1, we used an in vivo orthotopic mouse GBM model where we intracranially implanted either pcDNA3.1 or pcDNA3.1-ATIP1 stably transfected LNT-229 cells. Mice developing ATIP1 overexpressing tumors survived significantly longer (107.5 days) compared to the sham cohort (66 days; Figure 3g). Histological analyses demonstrated a significant reduction of tumor growth and proliferating cells in tumors of the ATIP1 cohort compared to the controls (Figure 3h).

### 2.4. ATIP1 Modulates ERK and AKT Activity

**Figure 4:**
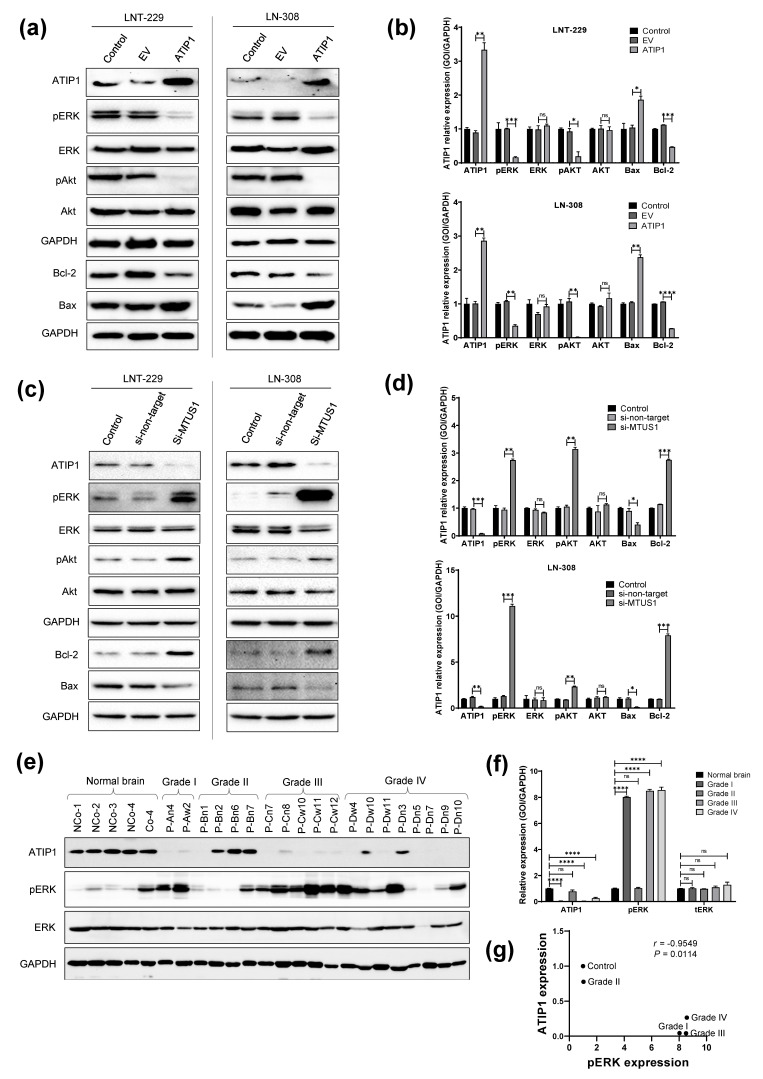
Enhanced expression of ATIP1 modulates the activity of glioma-associated signaling pathways. (**a**,**c**) Western blot of ATIP1, p-ERK, ERK, p-AKT, AKT, BAX and BCL-2 in parental (control), pcDNA3.1 (EV) and pcDNA3.1-ATIP1 (ATIP1) stably transfected LNT-229 and LN-308 cells (**a**) or 72 h after transient si-RNA transfection of parental LNT-229 and LN-308 cells. (**c**) GAPDH served as the loading control. One representative experiment out of three is shown. (**b**,**d**) Quantification of the above-mentioned proteins (n = 3, SEM; ns, * *p* < 0.05, ** *p* < 0.01, *** *p* < 0.001, **** *p* < 0.0001). (**e**) Western blot of ATIP1, p-ERK and ERK in glioma specimen (n = 3). (**f**) Quantification of the above-mentioned proteins in glioma specimen of different WHO grades (n = 3, ns, * *p* < 0.05, ** *p* < 0.01, *** *p* < 0.001, **** *p* < 0.0001). (**g**) Pearson parametric correlation test was used to analyze the significance between ATIP1 and p-ERK expression in glioma samples of different WHO grades.

Based on our data showing that ATIP1 mitigates proliferation and migration, we subsequently investigated its involvement in the activation of the cancer-associated ERK and AKT signaling pathways. In ATIP1 overexpressing LNT-229 and LN-308 cells, AKT and ERK1/2 phosphorylation, as well as phosphorylation of the ERK downstream target p90RSK, were reduced (Figure 4a,b, Figure S6). Additionally, pro-apoptotic BAX was up-and anti-apoptotic BCL-2-downregulated. To validate whether ATIP1 directly influences the activation of the above pathways and the expression of BAX and BCL-2, we knocked down *MTUS1* by siRNA. Indeed, reduced ATIP1 expression increased phospho-ERK1/2, phospho-AKT and also BCL-2, but reduced BAX (Figure 4c,d). We also investigated human glioma tissue for phospho-ERK and usually found lower amounts of phospho-ERK in those samples that express ATIP1 (Figure 4e,f). Pearson’s correlation analysis represents that ATIP1 and phospho-ERK expression is negatively correlated in clinical glioma specimens (Figure 4g).

### 2.5. ATIP1 Expression Is p53 Dependent and Modulates Irradiation-Induced Growth Reduction

**Figure 5:**
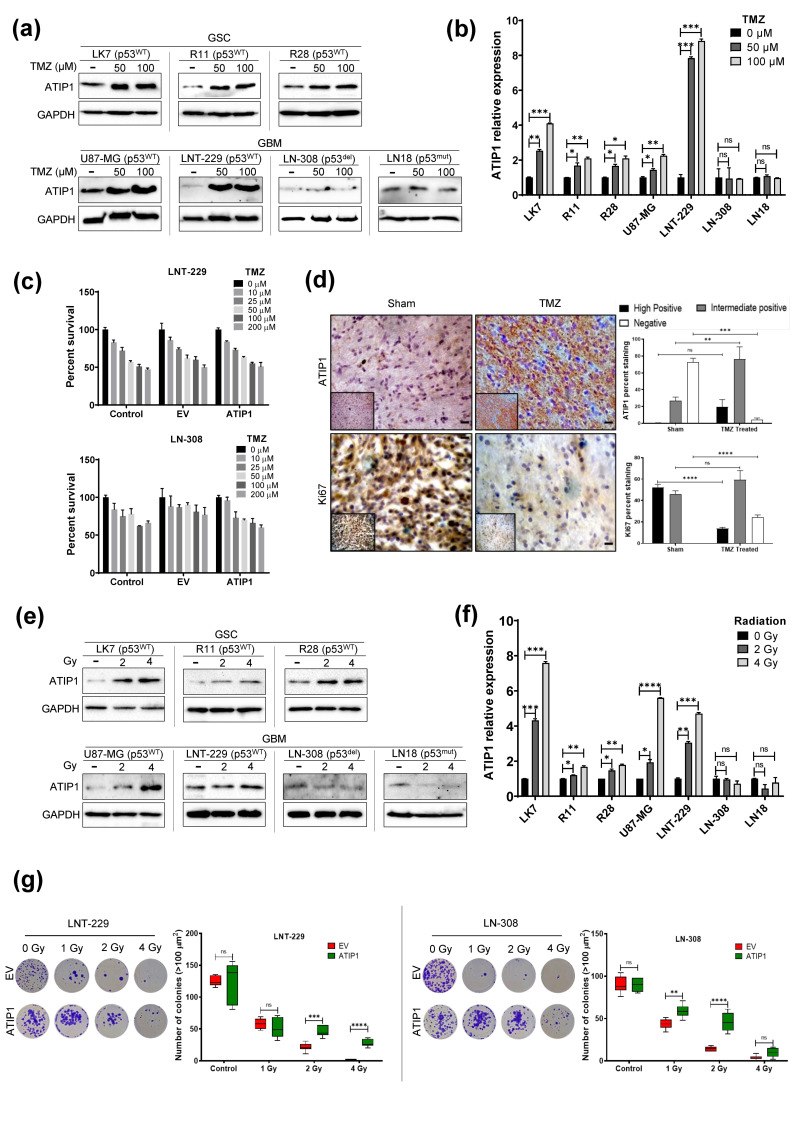
ATIP1 expression is modulated by TMZ and irradiation. (**a**) GBM and GSC cells were treated with TMZ and assessed for ATIP1 protein expression 72 h later. GAPDH serves as loading control (n = 3, one representative experiment is shown). (**b**) Quantification of protein expression after TMZ treatment as indicated in (**a**) (n = 3, SEM; ns, * *p* < 0.05, ** *p* < 0.01, *** *p* < 0.001, **** *p* < 0.0001). (**c**) Clonogenic survival of TMZ treated parental (control), pcDNA3.1 (EV) and pcDNA3.1-ATIP1 (ATIP1) stably transfected LNT-229 (upper panel) and LN-308 cells (lower panel, n = 3, SEM). (**d**) ATIP1 and Ki67 expression determined by immunohistochemical staining in sham (n = 6) or TMZ treated rats (n = 6) harboring C6 glioma (scale = 100 µm). The right panel shows the quantification of these proteins. (**e**). The cells were irradiated with 0 (-), 2 or 4 Gy. ATIP1 protein expression was analyzed 24 h later. GAPDH serves as loading control. (**f**) Quantification of ATIP1 expression in irradiated cells as indicated in (**e**) (n = 3, SEM; non-significant: ns, * *p* < 0.05, ** *p* < 0.01, *** *p* < 0.001, **** *p* < 0.0001). (**g**) pcDNA3.1 (EV) or pcDNA3.1-ATIP1 (ATIP1) stably transfected LNT-229 (left panels) and LN-308 (right panels) were irradiated with 0, 1, 2 or 4 Gy and clonogenic survival was analyzed. The first and third panels exemplarily show the outgrowth of cell clones, the graphs represent the total number of clones (n = 3, SEM; non-significant: ns, ** *p* < 0.01, *** *p* < 0.001, **** *p* < 0.0001).

Our data and a previous study identified ATIP1 as TSG that regulates the expression of *MMS2* [28]. Besides, in addition to the downregulation of *MTUS1* in HGG and recurrent gliomas, *MMS2* is also downregulated in these tumors (Figure 1c). As *MMS2* is a member of the postreplication repair pathway (PRRP), we suggest that, by modulating DNA-repair mechanisms, ATIP1 might interfere with glioma therapy like irradiation or TMZ-based chemotherapy. After TMZ treatment we observed elevated ATIP1 levels only in p53^WT^ but not in p53^mut^ or p53^del^ GBM cells (Figure 5a,b). To validate the role of p53 in the induction of ATIP1, LNT-229 p53^ts^ cells were subjected to 32.5 °C (p53^WT^) or 38.5 °C (p53^mut^). A significant increase in ATIP1 was detected only if p53^WT^ was present (Figure S7a,b). Additionally, we irradiated parental LNT-229 cells and observed that the upregulation of ATIP1 was accompanied by the upregulation of p53 (Figure S7c,d), indicating that p53 is an inducer of ATIP1 also in glioma cells.

However, even if ATIP1 was upregulated by TMZ, its elevated expression did not change the clonogenic survival of TMZ-treated LNT-229 (p53^WT^) and LN-308 (p53^del^) cells (Figure 5c) indicating that TMZ-induced DNA damage repair is not influenced by ATIP1. This also suggests that TMZ, not only by inducing DNA damage but also by upregulating ATIP1 in a p53-dependent manner, overwhelms the growth of cells, which under normal conditions express only low levels of ATIP1 and grow faster than cells that express high levels of ATIP1 (Figure 3a). TMZ-mediated ATIP1 upregulation in vitro reflects into the in vivo findings since in rats harboring p53^WT^ C6 glioma (Figure 5d), ATIP1 levels were elevated in the TMZ cohort. The TMZ-based ATIP1 upregulation was paralleled by reduced Ki67 expression, indicating a lesser proliferation rate in these tumors (Figure 5d, Tables S7 and S8).

As observed for TMZ, ATIP1 was also upregulated by irradiation in p53^WT^, but not in p53^del^ or p53^mut^ GBM cells (Figure 5e,f). Contrarily to TMZ, elevated ATIP1 levels interfered with the irradiation-mediated inhibition of clonogenic survival both in LNT-229 and LN-308 glioma cells (Figure 5g). A significant increase in cell viability in ATIP1 overexpressing glioma cells after irradiation represents that elevated ATIP1 levels lead to elevated survival (Figure S8). To determine whether the radio-resistance we observed in the ATIP1 overexpressing cells in vitro reflects the in vivo situation, we used the R2-database and correlated *MTUS1* mRNA expression and survival in patient cohorts that did not or received radiotherapy. Whilst in GBM patients elevated *MTUS1*/ATIP1 expression in the tumor tissue generally correlated with better survival (Figure 1h, Figure S2a), in the cohort of irradiated patients possessing tumors with higher *MTUS1* expression, this was slightly but not significantly associated with a worse outcome (Figure S9).

### 2.6. ATIP1 Promotes DNA Double-Strand Break (DSB) Repair

**Figure 6:**
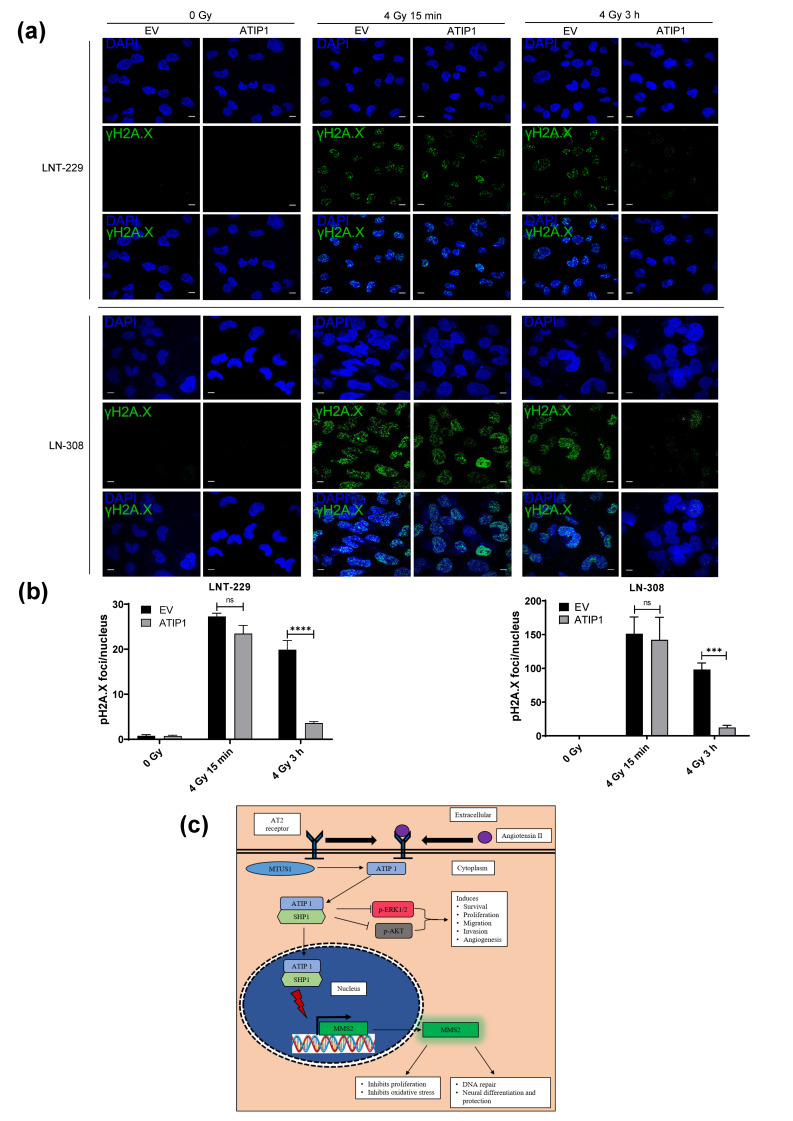
In glioma cells ATIP1 expression enhances the repair of irradiation-induced DNA damage. (**a**) γH2A.X foci formation in control (0 Gy) and irradiated (4 Gy) pcDNA3.1 (EV) and pcDNA3.1-ATIP1 (ATIP1) stably transfected LNT-229 and LN-308 cells at the illustrated time points (scale = 100 μm). (**b**) Quantification of γH2A.X foci per nucleus (n = 9, SEM; ns: not significant, *** *p* < 0.001, **** *p* < 0.0001). (**c**) A simplified model of the role of ATIP1 in tumor progression.

ATIP1 overexpression protected both LNT-229 and LN-308 cells from irradiation-induced cell death and growth inhibition but did not affect TMZ-mediated cytostatic effects (Figure 5c,g). In contrast to TMZ that produces the O6-methylation of guanines, amongst other damages, irradiation produces DSB. To investigate the dynamics of DNA damage and ATIP1 expression, LNT-229 and LN-308-control or ATIP1 overexpressing cells were irradiated and were stained for phosho^S139^-H2A.X (referred to as γH2A.X), a biomarker for DSB. γH2A.X foci were detectable 15 min post-irradiation in both control and ATIP1 overexpressing LNT229 and LN-308 cells. However, independent of p53, γH2A.X foci were nearly absent in ATIP1 overexpressing LNT-229 and LN-308 cells 3 h post-irradiation, whilst they are only slightly reduced in the sibling control cells (Figure 6a,b), indicating that ATIP1 boosts DSB repair. To evaluate whether the reduction of γH2A.X foci was linked to the p53 mediated induction of ATIP1, we irradiated LNT-229 cells and found that ATIP1 expression was increased 15 min after irradiation, this elevated expression being detectable also 3 h later (Figure S7c,d), which is the time point we detected a reduction in the number of foci also in control cells (Figure 6b). As a control for DNA repair, we also irradiated LNT-229-p53^ts^ cells at 32.5 °C (p53^WT^) or 38.5 °C (p53^mut^) and found a significant decrease in the number of foci 3 h after irradiation when cultivating the cells at 32.5 °C (Figure S10). This indicates that ATIP1 expression drives DSB repair, consequently interfering with the irradiation-induced reduction of clonogenic outgrowth. Additionally, a prolonged and p53-independent elevated expression of ATIP1 further strengthened this effect, leading to radiation resistance.

## 3. Discussion

Loss of function mutations in TSG has been identified in various cancers including lung, ovarian, pancreatic, uterine, head and neck, breast and bladder cancer [14,15,29,30]. *MTUS1*/ATIP1 has been reported as a TSG in a variety of cancers as it inhibits ERK phosphorylation, proliferation, tumor cell motility and metastasis [16]. However, there is no information about *MTUS1* being an important TSG also in glioma. In this study, we discovered a significant downregulation of ATIP1 and its up-and downstream partners *SHP1* and *MMS2* in glioma patients. Downregulation of these genes correlated with glioma progression since lower *ATIP1*, *SHP1* and *MMS2* levels were detected along with the downregulation of ATIP1. Whilst there seemed to be no correlation of ATIP1 expression and the IDH status of either LGG and HGG (Figure S1b), there is a strict correlation of ATIP1 expression with glioma malignancy since ATIP1 is significantly downregulated in glioma cell lines compared with non-neoplastic cells of brain origin (Figure 1j), in HGG compared with LGG (Figure 1f), in pilocytic astrocytomas compared to diffuse glioma (Figure 1d) as well as in recurrent gliomas compared with first time diagnosed gliomas (Figure 1e). Additionally, at least in GSC and glioma cell lines, we detected lower ATIP1 expression in those cells harboring an unmethylated MGMT promoter, which means that in those cells, due to enhanced repair of O6-methylguanines by MGMT, are not very vulnerable towards TMZ treatment, reflecting a more malignant type of glioma cells. Mining of the R2 database revealed that an elevated ATIP1 expression seems to be correlated with glioma patient survival. However, in this database, a small number of patients were analyzed for ATIP1 expression and a larger cohort is required to provide clear information. (Figure 1h). In the cohort of glioma, we received from the KIMS hospital in India, only a slight trend towards a prolonged survival of ATIP1^high^ patients was visible (Figure S2), which might also be due to the fact that in our cohort of 76 glioma only 6 tumors were classified as ATIP1^high^ tumors. Additionally, in the group of elderly that mainly harbor HGG, no patients with ATIP1^high^ expression were present (Figure S2). In summary, our data allude to the fact that ATIP1 expression is correlated with the grade of glioma malignancy since ATIP1 was reduced or even absent in HGG. This suggests that ATIP1 serves as a TSG also in glioma.

The function of ATIP1 as a TSG in glioma is substantiated by our observation that in glioma cells, elevated ATIP1 expression mitigates proliferation and clonogenic outgrowth as well as migration and invasion (Figure 3a–f) as has been demonstrated for several other tumors [31,32,33]. ATIP1 dramatically reduced glioma cell motility and enhanced cell adhesion at least partially by the construction of LFACs (Figure 3e,f). The ATIP1-mediated inhibition of proliferation and motility of glioma cells seems to be regulated by ERK and AKT since phosphorylation of ERK1/2 and AKT was strictly inhibited in ATIP1 cells, but was dramatically upregulated after knocking down *MTUS1* (Figure 5a,b). Upregulation of E-cadherin, a protein that strengthens cell–cell interactions, accompanied by the downregulation of MMP2, necessary to destroy the extracellular matrix, in ATIP1 overexpressing cells strengthen these findings since both proteins are downstream targets of the above-mentioned pathways (Figure 3d) [34,35,36,37].

The expression of TSG is a highly regulated process, and often TSGs are shut down by promoter-hypermethylation [27,38,39]. DNA-methylation near promoter regions play a crucial role [40], but this varies considerably in different cell types [41]. In this regard, methylation also at CpG sites close to TSS are negatively correlated with gene expression [42,43,44]. In glioma cells, the impact of DNA methylation on ATIP1 expression was shown by decitabine treatment, which leads to elevated ATIP1 levels in all glioma cells and low passage GSC (Figure 2c–e). The distal part of the CpG island represents a significantly higher methylation pattern in GSC and GBM cells, but not in non-neoplastic cells, whereas the methylation grade of the proximal CpG island was more variable, suggesting that the distal part is the more methylation prone part of the *MTUS1* 5′ CpG island. Unfortunately, we were not able to examine methylation of the most proximal 200 bp of the *MTUS1* CpG island. Therefore, one could speculate that methylation in this region might also provide an impact on ATIP1 expression and might also be responsible for differences in the ATIP1 levels we observed in tumor cells and tissue (Figure 1a–c,f). Although, we suggest that methylation of the proximal CpG island seems to be mainly responsible for *MTUS1* downregulation in HGG cells (LK7, LK31, R11 and R28) that provided the lowest ATIP1 levels and exhibited the highest methylation in this region (Figure 2b).

Many genes involved in the control of DNA repair are regulated by p53. The *MTUS1* promoter contains two p53 responsive elements [22]. Our study for the first time showed that TMZ or irradiation of GBM cells induces ATIP1 expression in a p53-dependent manner (Figure 5, Figure S7a). By ATIP1 upregulation, p53 might also be involved in the crosstalk between EGF and AT_2_R signaling as the group of Nouet observed a trans-inactivation of receptor tyrosine kinases by ATIP1 [23]. Nouet’s data have been refuted by our data since we observed a reduction of phospho-ERK also in p53^del^ LN-308 ATIP1 overexpressing cells. However, our study showed for the first time that ATIP1 inhibits not only EGFR/ERK signaling, but also blocks AKT activation (Figure 4a–d). AKT is a prominent modulator of the cytoskeleton [45], and therefore reduced phospho-AKT in ATIP1 overexpressing glioma cells might explain the observed mitigated migration, invasion and lesser lamellipodia, as well as the enhanced attachment of these cells (Figure 3e,f). Additionally, by modulating the activity of the above-mentioned signaling pathways, ATIP1 is able to regulate survival by modulating the expression of the apoptotic BAX and anti-apoptotic BCL-2 genes (Figure 4a–d).

ATIP1 expression in general correlated well with the prognosis of GBM patients (Figure 1h, Figure S2a), which has been buttressed by our orthotopic GBM mouse model (Figure 3g). However, ATIP1 regulates DNA-repair via *MMS2* [26], and this might interfere with the growth inhibitory effects of glioma therapy. Whilst elevated ATIP1 levels did not influence the effect of TMZ (Figure 5c), it promoted the clonogenic survival of irradiated cells independent of p53 (Figure 5g). Similar effects were observed in an irradiated GBM patient cohort where elevated ATIP1 levels were associated with a worse outcome (Figure S9). The ATIP1-mediated, irradiation protective effects in GBM cells seemed to be provoked by an elevated DSB repair as shown by reduced γH2A.X foci in ATIP1 overexpressing cells after 3 h (Figure 6b,c). Although, whether the enhanced DSB repair we observed was exclusively evoked by ATIP1 is debatable. It has been shown that *MMS2* and the ubiquitin-conjugating enzyme E2 13 (UBC13) form a stable complex, which is required for a p53-mediated DNA damage response [46]. Nevertheless, we observed this irradiation protective effect of ATIP1 also in p53^del^ LN-308 glioma cells, suggesting an additional p53-independent role of ATIP1 in DSB repair. However, to clarify the role of ATIP1 and its putative interference in DSB DNA damage-inducing therapies in GBM, further detailed investigations are needed.

## 4. Materials and Methods

### 4.1. Clinical Sample Collection and Processing

The study was carried out in accordance with the declaration of Helsinki and authorized by the Institutional Ethics Committee (IEC; Ref. No. UH/IEC2016/180; Date: 17/02/2016). Informed written consent was obtained from all participants. The surgically resected glioma samples were collected from the Krishna Institute of Medical Science (KIMS, Hyderabad, India). Patients diagnostics were confirmed clinically and histopathologically at the pathology facility of KIMS Hospital, and classification of samples was done according to the WHO classification [47]. For further detail refer to Table S1. The samples were snap-frozen and kept at −80 °C for further analysis. Normal brain tissue samples were collected from the National Institute of Mental Health and Neurosciences (NIMHANS), Bengaluru, India. The specimens consisted of 4 normal brain samples along with 4 epileptic brain samples, 6 WHO grade I pilocytic astrocytomas, 27 WHO grade II, 20 WHO grade III and 23 WHO grade IV glioma. Amongst these, 2 patients in grade I, 4 patients in grade II, 5 patients in grade III and 7 patients in grade IV were classified as recurrences. The clinical details of glioma specimen were provided by KIMS pathology department. None of the patients had received pre-operative chemotherapy, radiotherapy and targeted therapy except recurrent glioma patients. The samples were further processed for RNA and protein isolation and analysis of ATIP1 expression and its molecular correlations.

### 4.2. RNA Isolation, PCR and Quantitative Real-Time PCR (qRT-PCR)

For quantitative analyses, reverse transcription-polymerase chain reaction (RT-qPCR), RNA from either cells or tissue was isolated using Trizol (Sigma-Aldrich, MO, USA) and transcribed into cDNA using Superscript III (Invitrogen, Karlsruhe, Germany). RT-qPCR was performed using SYBR green master mix (Takara Bio, Mountain View, CA, USA) on an ABI7500 system (Applied Biosystems Incorporation, Darmstadt, Germany). Cycling conditions were as followed: 95 °C for 5 min, 40 cycles at 95 °C for 15 s, 60 °C for 1 min and 72 °C for 30 s. Relative mRNA expression was quantified, E^ΔΔCT^ (gene of interest)/E^ΔΔCT^ (housekeeping gene). Primers used for these analyses are shown in Table S3.

### 4.3. Western Blotting

The general procedure has been described previously [48]. Briefly, the cells were lysed in lysis buffer (Cell Signaling Technology Europe, Frankfurt/Main, Germany) + 1mM Phenylmethanesulfonylfluoride, passed through a 27-gauge syringe and clarified by centrifugation. Protein contents were analyzed according to Bradford (Sigma-Aldrich, Taufkirchen, Germany). The following primary antibodies (1:1000) were used: ATIP1 (Bioss, Woburn, MA, USA), phospho-ERK (Cell Signaling Technology Europe, Frankfurt/Main, Germany)), ERK, phospho-AKT, AKT, Bax, Bcl-2, E-cadherin, MMP-2, p53 (Santa Cruz Biotechnology, Dallas, TX, USA). Protein expression was imaged and quantified using the ChemiDoc MP system and Image Lab Software (Bio-Rad, Munich, Germany).

### 4.4. Histology and Immunohistochemistry

Rodent brain tissues were fixed with 4% PFA for 24 h, subjected to dehydration using 20 and 30% sucrose solutions and cryo-sectioned for histological analysis using a Leica Cryomicrotome CM3050S (Leica, Wetzlar, Germany). The images were captured using an Axioplan 2 microscope and imaging software (Carl Zeiss, Oberkochen, Germany). For human samples formalin fixed paraffin-embedded tissue sections were deparaffinized. To block endogenous peroxidase activity, the sections were treated with 3% H_2_O_2_ in methanol for 5 min. For antigen retrieval, sections were pretreated by boiling in Tris-EDTA buffer pH 9 for 20 min, washed with phosphate-buffered saline (PBS), blocked with 2% BSA, immunostained using antibodies against ATIP1 (1:100; Bioss Woburn, MA, USA), Ki67 (1:100; Novus Biologicals, Centennial, CO, USA) and GFAP (1:100; Novus Biologicals), washed again and incubated with polydetector HRP label followed by incubation with diaminobenzidine (DAB; Bio SB, Santa Barbara, CA, USA). Images were taken using an Olympus microscope (Olympus, Tokyo, Japan). ImageJ IHC profiler plugin, which designates the pixel intensity range between 0 to 60 for high positive staining; 61 to 180 for intermediate positive staining; and 181 to 235 for the negative staining, respectively, was used for expression grading [49].

### 4.5. Cell Culture and Reagents

Human GBM cell lines LNT-229 (p53^WT^, IDH^WT^), LN-308 (p53^del^, IDH^WT^) and LN18 (p53^mut^, IDH^WT^) were kindly provided by N. de Tribolet (Lausanne, Switzerland), U87-MG (p53^WT^, IDH^WT^) cells were obtained from ATCC (Manassas, USA). LNT-229-p53^ts^ cells, expressing a p53 temperature-sensitive mutant (V135A), were grown at 38.5 °C to express mutant p53 or at 32.5 °C to express wild type p53 [50,51]. C6 (p53^WT^) rat glioma cells were obtained from NCCS, Pune, India. The cells were maintained in DMEM (Sigma-Aldrich, Taufkirchen, Germany) containing 10% fetal bovine serum (FCS), penicillin (10,000 U/mL) and streptomycin (100 mg/mL; ThermoFisher Scientific/Gibco, Karlsruhe, Germany). Low passage GSC, R11 (p53^WT^, IDH^WT^), R28 (p53^WT^, IDH^WT^) were a kind gift from C. Beier (University of Regensburg) [2]. The above-mentioned cell lines have been sent for authentication to the DSMZ German Collection of Microorganism and Cell Cultures in 2012. Thereon, the cells were frozen at −145 °C in individual labelled and electronically documented vials. LK7 (p53^WT^, IDH^WT^), LK28 (p53^WT^, IDH^WT^) and LK31 (p53^WT^, IDH^WT^) low passage GSC, derived from patients with primary GBM, were obtained from L. Klumpp and S. Huber (Department of Radiation Oncology, University Hospital Tübingen) and were maintained up to passage 30. Information about p53 and IDH mutations, as well as MGMT promoter methylation of all cell lines, are available in Table S2. R11, R28 and LK cells were maintained as spheres in stem cell permissive DMEM-F12 medium supplemented with 20 ng/mL of human recombinant epidermal growth factor (EGF; BD Biosciences, Heidelberg, Germany), human recombinant basic fibroblast growth factor (bFGF; R&D Systems, Wiesbaden, Germany), human leukemia inhibitory factor (LIF; Millipore, Billerica, MA, USA), 2% B27 supplement (Life Technologies, Carlsbad, CA, USA), penicillin (10,000 U/mL) and streptomycin (100 mg/mL, ThermoFisher Scientific/Gibco). Normal human astrocytes (NHA, passages 2 to 4, Lonza, Basel, Switzerland) were maintained in supplemented ABM medium (Lonza, Basel, Switzerland). Human brain microvascular endothelial cells (HBMvEC) obtained from iXCells (San Diego, CA, USA) were cultured in supplemented EGM-2 medium (Lonza). All cell lines (except LNT-229 p53^ts^) were maintained at 37 °C in a 5% CO_2_ humidified atmosphere. Periodically, all cell lines were tested to be mycoplasma free using the Lonza MycoAlert mycoplasma detection kit. Decitabine (5-Aza-2′-deoxycytidine: 5-Aza-dC; Sigma-Aldrich) treatment was performed in standard growth medium for 72 h with a repeated change of the medium every 24 h. To determine clonogenic survival, 1000 cells were seeded in 6 well plates. After approximately 10 doubling times the cells were fixed with 4% paraformaldehyde (PFA) and stained with crystal violet as previously described [52].

### 4.6. Plasmids and Transfection

To generate ATIP1 overexpressing glioma cells, ATIP1 (NCBI Reference Sequence: NM_020749.4) was amplified by PCR from cDNA of normal human brain tissue obtained from the National Institute of Mental Health and Neurosciences (NIMHANS), Bengaluru, India and was cloned into pCDNA3.1-Myc to express myc-tagged *MTUS1*/ATIP1 (for primers see Table S4). Glioma cells were transfected with pcDNA3.1-myc or pCDNA3.1-myc-ATIP1 using Effectene (Qiagen, Hilden, Germany) following the manufacturer’s instructions, positive cells were selected using G418.

### 4.7. CpG Island Identification, Bisulfite Conversion and Methylation Analysis

The CpG island in the 5′ TSS of the *MTUS1* coding region was identified using Methprimer software (Beijing, China). The criteria used for prediction were: Island size > 100, GC percent > 50.0, Obs/Exp > 0.6. Genomic DNA was isolated using the Qiagen DNA extraction kit (Qiagen, Hilden, Germany) and was subjected to bisulfite conversion using the DNA Lightening conversion kit (Zymo Research GmbH, Freiburg, Germany) according to the manufacture’s protocol. Bisulfite-converted DNA was used for amplification of the predicted *MTUS1* promoter region using hot start Platinum Taq DNA polymerase (ThermoFisher Scientific/Invitrogen, Karlsruhe, Germany) (Primer see Table S5). PCR fragments were cloned in the pCR2.1 vector using the TA-TOPO cloning kit (ThermoFisher Scientific/Invitrogen). Positive clones were sent for Sanger sequencing (Eurofins, Ebersberg, Germany) and the methylation pattern was analyzed using QUMA software (CDB, Riken, Japan). The raw fastq sequence files were uploaded on Sequence Read Archive (SRA, NCBI) server with accession ID: PRJNA633153.

### 4.8. Cell Migration, Invasion and Adhesion Assay

To determine the migration capability of cells, wound healing and Boyden chamber assays were used. For wound healing assays the cells were grown to about 80% confluency. To normalize the effect of proliferation in the analysis, the cells were precedingly treated with mitomycin A (2.5 µg/mL) for 3 h. After washing, a scratch was set using a pipet tip. Images were taken every 24 h. In the Boyden chamber assay, 2 × 10^4^ glioma cells were seeded in serum reduced DMEM (4% FCS) into either uncoated (migration, Corning, Amsterdam, Netherlands) or Matrigel-coated (invasion, Corning) 8 µm transwell inserts. As an attractant, NIH-3T3 cell-conditioned media was used. The plates were incubated at 37 °C for 20 (migration) or 36 h (invasion). The cells on the bottom part of the insert were fixed, stained with hematoxylin and counted using ImageJ software. To determine cell adhesion, the cells were seeded on poly-L-lysine coated coverslips, allowed to grow for 48 h and were then fixed with 4% PFA. Staining was accomplished using the Actin Cytoskeleton and Focal Adhesion Staining Kit (Millipore, Schwalbach, Germany), which allows the detection of actin filaments (by phalloidin), or focal contacts (by vinculin antibody) as well as of the nucleus (DAPI). Quantification of migration, invasion and adhesion (focal adhesion complexes and of vinculin-positive cells) was done using Image J software [53].

### 4.9. siRNA Transfection

To knockdown *MTUS1*, LNT-229 or LN-308 cells at 60–80% confluency were transfected with either 50 nM of control non-target or *MTUS1* siRNA mixes (Santa Cruz Biotechnology, Dallas, TX, USA) containing a mix of three to five different siRNAs using the Viromer Blue transfection reagents (Biontech, Mainz, Germany). Whole-cell lysates were prepared from the transfected cells 72 h later.

### 4.10. Animal Experiments

Rat experiments have been approved by the Institutional Animal Ethics Committee (IAEC), University of Hyderabad, India. For immunohistochemical analysis, 1 × 10^5^ C6 cells were orthotopically engrafted into the right striatum of Wistar rats under stereotactic guidance [48]. On day 7 after implantation, two rats were randomly selected, sacrificed and screened for the presence of tumors by H&E staining. Rats were randomly divided into sham (n = 6) or TMZ-treated (n = 6) cohorts and were intraperitoneally injected three times with TMZ (10 mg/kg) or saline containing 10% DMSO (sham) every second day as described [54]. Two weeks after tumor implantation the rats were sacrificed and fixed by PFA perfusion. Brain tissues were collected and used for histology and immunohistochemical analyses.

All mouse experiments were conducted following the appropriate guidelines approved by the regional board Tübingen (permit number N10-18G). For survival analyses, Rj:NMRI-Foxn1^nu/nu^ mice (Janvier, Le Genest-Saint-Isle, France) were orthotopically inoculated with 5 × 10^4^ LNT229-pcDNA3.1 (n = 11) or LNT-229-pcDNA3.1-ATIP1 stably transfected glioma cells (n = 11) into the right striatum as previously described [55]. The mice were sacrificed as soon as they developed neurological symptoms. Survival was determined by the generation of Kaplan–Meier survival curves (n = 8) and histological studies (n = 3) were performed as mentioned above. For immunohistochemical analyses, the mice were sacrificed three weeks after intracranial implantation of the tumor cells.

### 4.11. Radio-Sensitivity Analysis

To determine the effect of ATIP1 on the glioma cell sensitivity towards irradiation, ATIP1 overexpressing or EV control cells as well as LNT-229-p53^ts^ cells (grown at 32.5 °C or 38 °C) were irradiated using a Gammacell GC-40 exactor (Nordion, Ottawa, ON, Canada). To determine clonogenic survival, the cells were seeded as described above. Cell viability was assessed by incubating the cells in methyl thiazole tetrazolium (MTT, 5 mg/mL; Sigma-Aldrich, Taufkirchen, Germany) for 4 h at 37 °C, the formazan crystals thus formed were solubilized in DMSO and absorbance was measured at 570 nm. To investigate the dynamics of residual DNA double strand breaks after irradiation, the cells were stained using an anti-phospho-H2A.X (Ser139) antibody (Millipore, Schwalbach, Germany), counterstained with DAPI and were microscopically analyzed using a laser scan confocal microscope (Carl Zeiss LSM 510). Quantification of γH2A.X foci, representing DNA double strand breaks, was performed using Image J software.

### 4.12. Cell Cycle Analyses

To determine the percent of cells in each phase of the cell cycle (G1, S or G2/M), EV and ATIP1 stably transfected LNT-229 and LN-308 cells were seeded in 60 mm plates. After 48 h the cells were washed in PBS, trypsinized and washed again with PBS. The cells were fixed in 70% cold ethanol for 16 h, washed twice with PBS and flow cytometry buffer and incubated in dark for 30 min with propidium iodide (PI)/RNase A buffer (50 µg/mL PI, 100 µg/mL RNase A in flow cytometry buffer. The cells in different phases were analyzed using a CyAn ADP flow cytometer and FlowJo software (FlowJo LCC, BD Life Sciences, Heidelberg, Germany).

### 4.13. Data Acquisition and Statistical Analysis

For survival analysis in human cohorts, the “R2: Genomics Analysis and visualization” platform (https://hgserver1.amc.nl/cgi-bin/r2/main.cgi (accessed on 31 January 2021)) was used. All experiments were performed independently at least thrice unless mentioned otherwise. Statistical analysis was performed using multiple pairwise comparisons for in vitro data. Overall survival probability was analyzed by Kaplan–Meier survival analysis and Log-rank (Mantel–Cox) using GraphPad Prism 8. The Log-rank (Mantel–Cox) test was implemented for in vivo survival analysis. Statistical comparisons were made using the student’s two tailed-unpaired *t*-tests. Correlation analysis between p-ERK and ATIP1 relative expression was performed using the parametric Pearson’s test. Results are represented as mean ± standard error mean (SEM). *p*-values of <0.05 are considered as statistically significant (ns: not significant; * *p* < 0.05, ** *p* < 0.01, *** *p* < 0.001, **** *p* < 0.0001).

## 5. Conclusions

Although we have not explored the involvement of the complete renin-angiotensin system in our study, we showed that the *MTUS1*/ATIP1 serves as a TSG also in glioma. In this regard, ATIP1 provides multiple tumor-suppressive functions like mitigating proliferation, cell motility and clonogenic survival. Additionally, in glioma, *MTUS1*/ATIP1 is a prognostic marker since its expression correlated well with glioma malignancy and survival [17,32,56]. However, ATIP1 is an important player in DNA repair processes and this might interfere with DNA-damaging tumor therapies. In glioma cells, elevated ATIP1 levels push DNA repair and protect the cells from irradiation-induced DNA damage. Therefore, it should be kept in mind that in HGG patients that possess highly ATIP1 positive tumors, even being ATIP1-correlated with a better basal outcome, the elevated expression of ATIP1 might interfere with the anti-tumoral effects of irradiation.

## Data Availability

The data presented in this study are available on request from the corresponding authors.

## Supplementary Materials

The following are available online at https://www.mdpi.com/2072-6694/13/6/1245/s1, Table S1: Patient data, Table S2: p53, IDH and MGMT status of GSC and glioma cell lines, Table S3: Primer used for quantitative RT-PCR analyses, Table S4: Primer used to amplify ATIP1 cDNA, Table S5: Primer used for amplification of bisulfite-converted DNA, Table S6: Quantification of ATIP1 in clinical human glioma specimen (IHC), Table S7: Quantification of ATIP1 in rat brain cryosections (IHC), Table S8: Quantification of Ki67 in rat brain cryosections (IHC), Figure S1: Correlation of ATIP1 expression with IDH mutations and MGMT promoter methylation, Figure S2: Correlation analyses of age and gender-specific ATIP1 expression with glioma patient’s survival, S3: ATIP1 expression in control and ATIP1 stably transfected glioma cell lines., Figure S4: ATIP1 modulates cell cycle distribution and migration, Figure S5: Elevated ATIP1 expression reduces glioma cell migration and invasion, Figure S6: Phospho-p90^RSK^ expression is reduced in ATIP1 overexpressing glioma cells, Figure S7: Irradiation induces ATIP1 expression, Figure S8: Elevated ATIP1 expression enhances the survival of irradiated glioma cells, Figure S9: Correlation of ATIP1 expression with the survival of glioma patients receiving irradiation therapy (R2 database mining), Figure S10: p53 expression modulates DNA repair dynamics.
