# Supplementary material for: The Tumor Suppressor MTUS1/ATIP1 Modulates Tumor Promotion in Glioma: Association with Epigenetics and DNA Repair
Source: Cancers (Basel). 2021 Mar 12;13(6):1245. doi: 10.3390/cancers13061245 (PMC7999421; doi:10.3390/cancers13061245)
Supplement: Supplementary file 1 [file cancers-13-01245-s001.pdf]

cancers

MDPI

Electronic Supplementary Material

Article

The tumor suppressor *MTUS1*/ATIP1 modulates tumor promotion in glioma: association with epigenetics and DNA repair

Nikhil Ranjan [1,2], Vimal Pandey [1], Manas Panigrahi [3], Lukas Klumpp [4], Ulrike Naumann [2,†,*] and Prakash Babu Phanithi [1,†,*]

[1] Laboratory of Neuroscience, Department of Biotechnology & Bioinformatics, School of Life Sciences, University of Hyderabad, Telangana, India, 500046; nranjan03@uohyd.ac.in
[2] Hertie Institute for Clinical Brain Research, Tübingen Neurocampus (TNC), Molecular Neuro-oncology, University of Tübingen, Tübingen, Germany, 72076; ulrike.naumann@uni-tuebingen.de
[3] Department of Neurosurgery and Pathology, Krishna Institute of Medical Sciences (KIMS), Secunderabad, Telangana, India, 500003; manasp1966@gmail.com
[4] Radiation Oncology, University Hospital Tübingen, Germany, 72076; lukas.klumpp@uni-tuebingen.de
† These authors share equal senior authorship
* Correspondence: prakash@uohyd.ac.in; Tel.: +91-40-23134584 (P.P.B); ulrike.naumann@uni-tuebingen.de Tel.: +49-7071-2980707 (U.N.)

The supplementary material file includes:

Table S1: Patient data.
Table S2. p53, IDH and MGMT status of GSC and glioma cell lines.
Table S3. Primers used for quantitative RT-PCR analyses.
Table S4. Primers used to amplify ATIP1 cDNA**.**
Table S5. Primers used for the amplification of bisulfite-converted DNA.
Table S6. Quantification of ATIP1 in clinical human glioma specimen (IHC).
Table S7. Quantification of ATIP1 in rat brain cryosections (IHC).
Table S8. Quantification of Ki67 in rat brain cryosections (IHC).

Figure S1. Correlation of ATIP1 expression with IDH mutations and MGMT promoter methylation.
Figure S2. Correlation analyses of age and gender-specific ATIP1 expression with glioma patient´s survival
Figure S3. ATIP1 expression in control and stably transfected glioma cell lines.
Figure S4. ATIP1 modulates cell cycle distribution and migration.
Figure S5. Elevated ATIP1 expression reduces migration and invasion.
Figure S6. Phospho-$p90^{RSK}$ expression is reduced in ATIP1 overexpressing glioma cells.
Figure S7. Irradiation induces ATIP expression.
Figure S8. Elevated ATIP1 expression enhances the survival of irradiated glioma cells.
Figure S9. Correlation of ATIP1 expression with the survival of glioma patients receiving irradiation therapy (R2 database mining).
Figure S10. p53 expression modulates DNA repair dynamics.

1. SUPPLEMENTARY TABLES

Table S1: Patient data

Pseudony-mous data	Age	Sex	Classification	WHO grade	IDH	Relative ATIP1 expression	Overall survival (month)
P Aw1	24	M	PA	I	ND	0.339578176	52
P-Aw2	7	M	Rec. PA	I	ND	0.017531406	46
P-An1	4	M	PA	I	WT	1.283537063	50
P-An3	22	M	PA	I	ND	0.001814927	54
P-An4	15	M	Rec. PA	I	ND	0.055116452	38
P-An7	12	M	PA	I	ND	0.23842974	0.06
P-Bw1	30	M	AA	II	WT	0.056063496	54
P-Bw2	30	F	Rec. OA	II	Mut	0.051917368	36
P-Bw3	53	M	Diffuse A, NOS	II	Mut	0.387771193	47
P-Bn1	27	M	Rec. diffuse A	II	Mut	0.458630228	37
P-Bn2	40	F	A	II	Mut	0.86244398	ND
P-Bn3	45	M	Diffuse A	II	ND	0.019620155	49
P-Bn4	40	F	Diffuse A	II	Mut	0.211672108	36
P-Bn5	56	M	OD	II	ND	0.246692066	22
P-Bn6	25	F	A	II	Mut	1.491968347	39
P-Bn7	52	M	Diffuse A	II	WT	1.310189831	41
P-Bn8	41	M	OD, NOS	II	WT	0.066403866	52
P-Bn10	33	M	Diffuse A	II	Mut	0.048025681	40
P-Bn11	32	M	Anaplastic OD	II	Mut	0.061334734	40
P-Bn13	58	M	Low grade A	II	Mut	0.157209863	37
P-Bn14	24	F	Low grade A	II	WT	0.179462451	43
P-Bn15	60	M	OD, NOS	II	WT	0.073359725	42
P-Bn16	45	M	OA	II	WT	0.078293743	46
P-Bn17	33	M	OD	II	Mut	0.10123464	29
P-Bn18	16	M	OD	II	Mut	0.261554	55
P-Bn19	30	M	OD	II	Mut	0.104992646	42
P-Bn20	44	F	OD	II	WT	0,109845091	39
P-Cw1	47	M	Anaplastic OA	III	Mut	0.170218524	22
P-Cw2	46	M	AA	III	Mut	1.736959373	10
P-Cw3	18	F	Anaplastic OA	III	ND	0	23
P-Cw5	33	M	AA	III	ND	0.034349049	47
P-Cw6	16	M	Anaplastic OD	III	ND	0.039727551	3
P-Cw7	55	F	Anaplastic OA	III	Mut	0.03255926	51
P-Cw10	48	M	Anaplastic OD	III	Mut	0.010428393	41
P-Cw11	48	M	Anaplastic OD	III	WT	0.056572209	11
P-Cw12	56	M	Rec. diffuse AA	III	Mut	0.074458337	4

P-Cn1	40	F	AA	III	WT	0.042937733	4
P-Cn2	51	M	AA	III	Mut	0.04845835	46
P-Cn3	1	M	Anaplastic OA	III	WT	0.314522621	38
P-Cn4	59	M	Anaplastic OA	III	Mut	0.07555641	17
P-Cn5	34	F	AA, NOS	III	ND	0.031254369	48
P-Cn7	39	M	Rec. anaplastic OA	III	ND	0	ND
P-Cn8	24	F	Rec. AA	III	Mut	0.056572209	41
P-Dw1	40	M	GBM	IV	WT	0.032029361	40
P-Dw2	47	M	GBM, NOS	IV	ND	0.557466537	55
P-Dw3	58	M	Rec. GBM	IV	WT	0.000381637	21
P-Dw4	24	F	GBM	IV	ND	0.000489013	39
P-Dw5	50	M	GBM and OD	IV	Mut	0.038232658	36
P-Dw6	52	M	GBM	IV	Mut	0.011214158	9
P-Dw7	11	M	GBM	IV	WT	0.201158192	36
P-Dw8	59	M	GBM, NOS	IV	Mut	0.010705188	14
P-Dw9	60	F	Rec. GBM, NOS	IV	WT	0	12
P-Dw10	55	M	GBM	IV	WT	1.094447696	10
Dw11	78	F	GBM	IV	Mut	1.078E-05	17
P-Dn2	67	M	GBM	IV	Mut	0.019469398	28
P-Dn3	49	M	GBM	IV	WT	1.197526875	49
P-Dn4	52	F	GBM, NOS	IV	WT	0.008483491	34
P-Dn5	62	M	Rec. GBM	IV	WT	0.000372926	36
P-Dn7	67	M	GBM	IV	Mut	0.016799674	30
P-Dn8	65	M	GBM	IV	WT	0.060260604	46
P-Dn9	40	M	GBM	IV	Mut	0	33
P-Dn10	41	F	Rec. GBM	IV	Mut	0	43
P-Bn21	40	M	Rec. OA	II	WT	0.005456305	30
P-Bn22	48	M	Rec. OD	II	WT	0.002886281	30
P-Bn23	52	F	Diffuse AA	II	Mut	0.020310731	37
P-Bn24	34	M	OA	II	Mut	0.005979648	30
P-Bn25			OA	II	Mut	0.007569845	28
P-Bn26	68	F	OD	II	ND	0.007428301	42
P-Cn9	31	F	Rec.AA	III	ND	0.003736537	16
P-Cn10	12	F	Rec.AA	III	Mut	0.007099354	10
P-Cn11	55	M	Anaplastic OA	III	WT	0.000325399	42
P-Cn12	42	F	Anaplastic OD	III	Mut	0.002698785	32
P-Dn11	31	F	Rec. GBM	IV	Mut	0.085655136	30
P-Dn12	69	M	GBM	IV	Mut	0.000150811	25
P-Dn17	37	M	Rec. GBM	IV	WT	0.004728046	27
P-Dn18	78	M	Rec. GBM	IV	WT	0.04291154	8

A: astrocytoma; AA: anaplastic astrocytoma, F: female; GBM: glioblastoma M: male; OA: oligoastrocytoma; OD: oligodendroglioma; NOS: not other specified, ND: not determined; mut: mutated; PA: pilocytic astrocytoma; Rec. Recurrent; WT: wild type; Mut: mutant. The IDH status was analyzed by PCR and immunohistochemistry.

Table S2. p53, IDH and MGMT status of glioma cell lines [1-5]

Cells	p53	IDH	MGMT
LK7	WT	WT	u
LK28	WT	WT	m
LK31	WT	WT	m
R11	WT	WT	m
R28	WT	WT	u
LN-18	mut.	WT	u
LNT-229	WT	WT	m
LN-308	del.	WT	m
U87-MG	WT	WT	m

WT: wild type; mut: mutated; del: deleted; u: unmethylated; m: methylated

Table S3. Primers used for quantitative RT-PCR analyses

Primer	Sequence (5′-3′)
ATIP1 Forward	GGAGAGCTAGTCACTGCTTCAACC
ATIP1 Reverse	GTCCCGAAGCTTTTCATACTCCC
SHP1 Forward	GGAGTCGGAGTACGGGAACAT
SHP1 Reverse	ATCCTCCTTGTGTTTGGACGA
MMS2 Forward	ATTGGGCCACCAAGGACAAA
MMS2 Reverse	TAACACTGGTATGCTCCGGG
GAPDH Forward	TGACCCCTTCATTGACCTCA
GAPDH Reverse	GAGATGATGACCCTTTTGGCT

Table S4. Primers used to amplify ATIP1 cDNA

Primer	Sequence (5′-3′)
ATIP1 Forward	CGGATCCATGTTGTTGTCTCCCAAATTC
ATIP1 Reverse	GCTCGAGTCATCTGGGTGAAATGCTG

Table S5. Primers used for the amplification of bisulfite-converted DNA

Primer	Sequence (5′-3′)
Distal region-Forward	GAGGTAAGTAGGAGTTAGATTTTTTATT
Distal region-Reverse	AAAAAACAACCCTCAAAAAACCTAAAT
Proximal region-Forward	GATGGTGGTTGGTTTTGGTTTTT
Proximal region-Reverse	CACTTACCCACAACTCCTTCAAA

Table S6. Quantification of ATIP1 in clinical human glioma specimen (IHC)

	High positive	Intermediate positive	Negative
Normal brain	20.27 ± 3.811	75.94 ± 8.261	3.79 ± 1.594
Grade I	4.78 ± 0.3230	60.61 ± 0.7134	34.61 ± 0.09205
Grade II	4.65 ± 1.316	60.87 ± 3.781	34.48 ± 0.9083
Grade III	2.90 ± 0.5364	57.29 ± 8.805	39.81 ± 8.268
Grade IV	0.98 ± 0.1096	31.956 ± 0.7717	67.07 ± 0.6556

Staining intensity of ATIP1 in human glioma specimen of different WHO grades. Staining intensity (% staining of samples) was graded to be either highly positive, intermediate positive or negative as described in the methods part (normal brain n=4, WHO grade I n=4, WHO grade II n=12, WHO grade III n=18, WHO grade IV n=15)

Table S7. Quantification of ATIP1 in rat brain cryosections (IHC)

	High positive	Intermediate positive	Negative
Sham-treated	0.311 ± 0.311	26.835 ± 4.273	72.854 ± 4.584
TMZ-treated	19.390 ± 8.835	76.397 ± 14.492	4.213 ± 2.159

Staining intensity of ATIP1 in either sham or TMZ-treated rats harboring C6 GBM. Staining intensity (% staining of samples) was graded to be either highly positive, intermediate positive or negative as described in the methods part (sham n=6, TMZ n=6). For further information please refer to Fig. 5.

Table S8. Quantification of Ki67 in rat brain cryosections (IHC)

	High positive	Intermediate positive	Negative
Sham-treated	52.41 ± 2.596	47.59 ± 4,0637	0 ± 0
TMZ-treated	13.65 ± 1.367	59.26 ± 8,735	27.09 ± 2.369

Staining intensity of Ki67 in either sham or TMZ-treated rats harboring C6-GBM. Staining intensity (% staining of samples) was graded to be either highly positive, intermediate positive or negative as described in the methods part (sham n=6, TMZ n=6). For further information please refer to Fig. 5.

2. SUPPLEMENTARY FIGURES

Figure S1

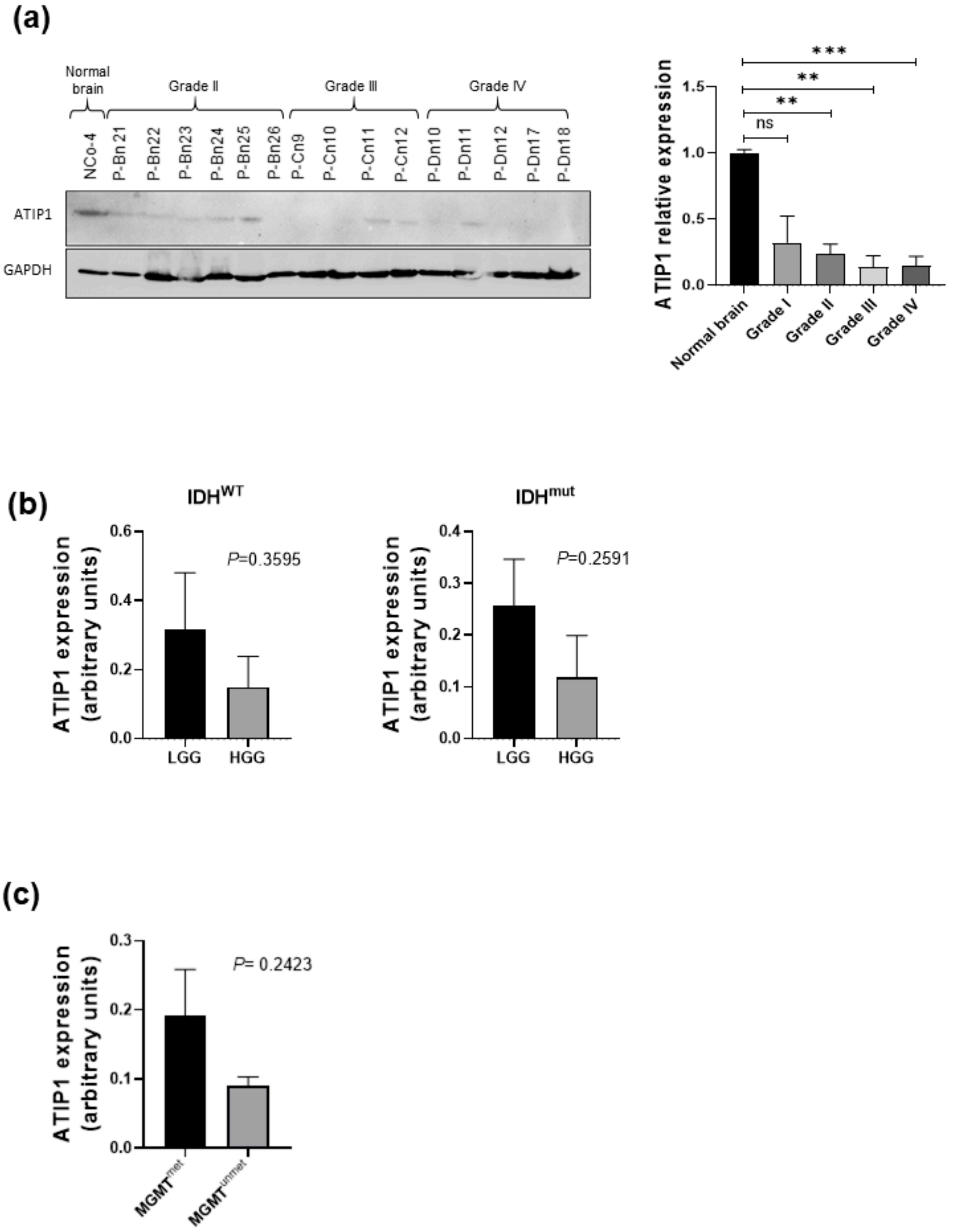

Figure S1. Correlation of ATIP1 expression with IDH mutations and MGMT promoter methylation. a. ATIP1 immunoblot of additional glioma specimen of different WHO grades and normal brain. One representative immunoblot is shown. The right graph shows the mean expression of ATIP1 in glioma compared to normal brain tissue (normal brain, n=4; grade I, n=6; grade II, n=27; grade III, n=20; grade IV, n=23). b. ATIP1 expression in IDH^{WT} LGG (n=10); HGG (n=12) and IDH^{mut} LGG (n=15); HGG (n=21) specimen. c. ATIP1 expression in MGMT methylated (n=6) and MGMT unmethylated promoter (n=3), patient-derived GSC and established glioma cell lines.

Figure S2

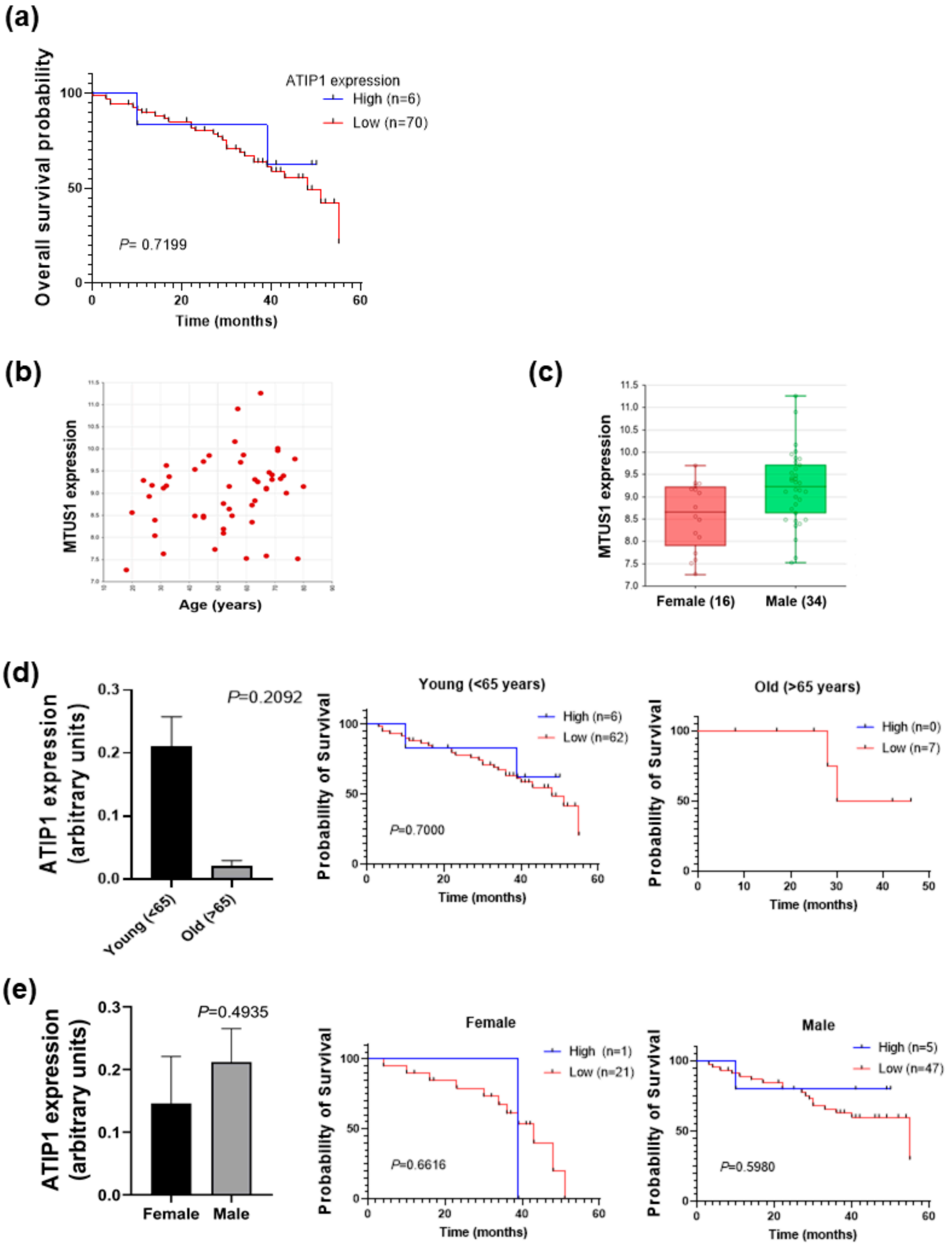

Figure S2. Correlation analyses of age and gender-specific ATIP1 expression with glioma patient´s survival. a. Overall survival analysis of the Indian cohort of glioma patients comparing ATIP1^{high} (n=6) and ATIP1$^{intermediate/low}$ patients (n=70, Log-Rank Mantel-Cox test). b. Correlation of *MTUS1*/ATIP1 expression with the age of glioma patients, data were extracted using the R2 database. c. Gender-related expression of *MTUS1*/ATIP1 in glioma patients, data were extracted from the R2 database. d. ATIP1 expression in younger (<65) and older (>65) glioma patients (left panel). The middle and right panels present the survival of younger and older patients in comparison with, ATIP1 expression, data were analyzed from the Indian glioma patient cohort. e. ATIP1 expression in male and female glioma patients (left panel). The middle and right panels present the survival of female and male glioma patients in comparison with ATIP1 expression, data were analyzed from the Indian glioma patient cohort.

Figure S3

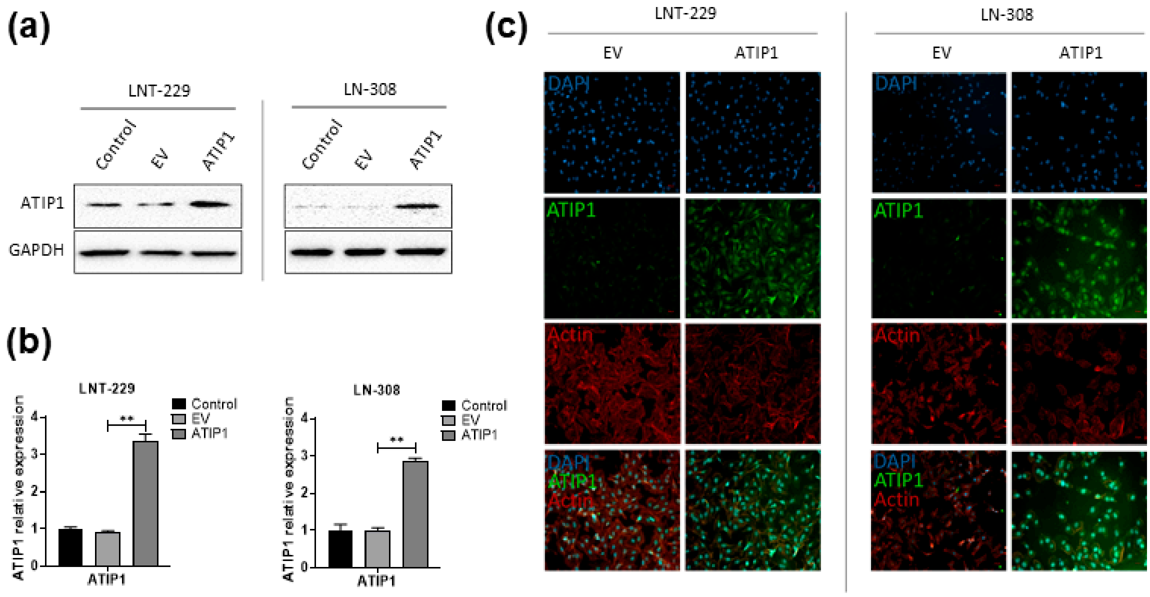

Figure S3. ATIP1 expression in control and stably transfected glioma cell lines. a. Immunoblot of whole-cell lysates derived from either LNT-229 and LN-308 parental (control) or stably transfected cells using the empty vector pcDNA3.1 (EV) or pcDNA3.1-ATIP1 (ATIP1). b. Quantification of ATIP1 as indicated in (a) (n=3, SEM, ** *P* < 0.01). c. Immunofluorescence staining of ATIP1 in EV and ATIP1-stably transfected LNT-229 and LN-308 cells (bar = 50 µm).

Figure S4

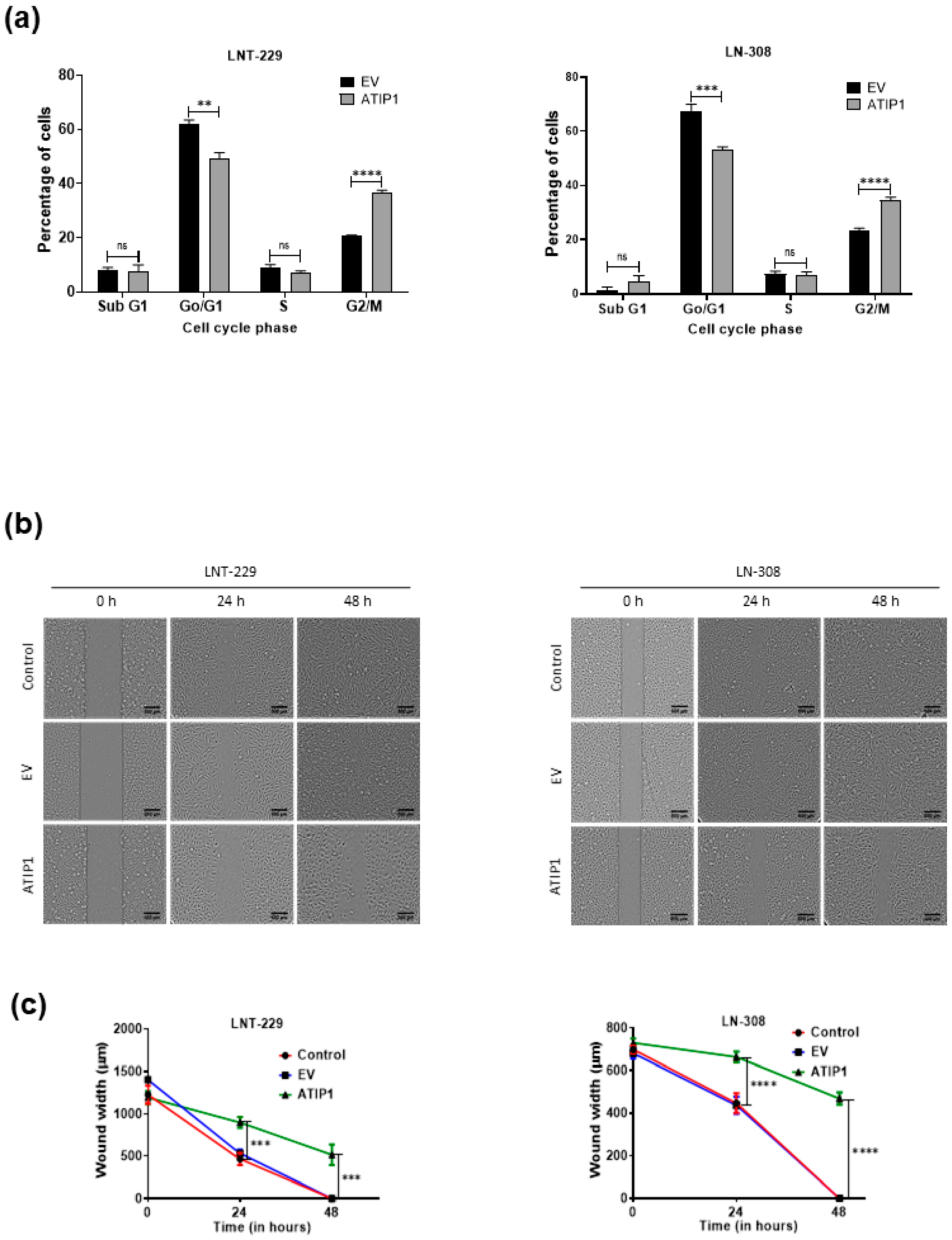

Figure S4. ATIP1 modulates cell cycle distribution and migration. a. Cell cycle analysis displaying different phases of cell cycle in pCNA3.1 (EV) and pcDNA3.1-ATIP1 (ATIP1) stably transfected LNT-229 and LN-308 cells (n=3, SEM; ns, *** $P < 0.001$, *** $P < 0.001$, **** $P < 0.0001$). b. Wound healing scratch assay of parental (control), pcDNA3.1 (EV) and pcDNA3.1-ATIP1 (ATIP1) stably transfected LNT-229 and LN-308 cells (bar = 500 µm). c. Quantification of wound closure as indicated in b. (n=3, SEM; *** $P < 0.001$).

Figure S5

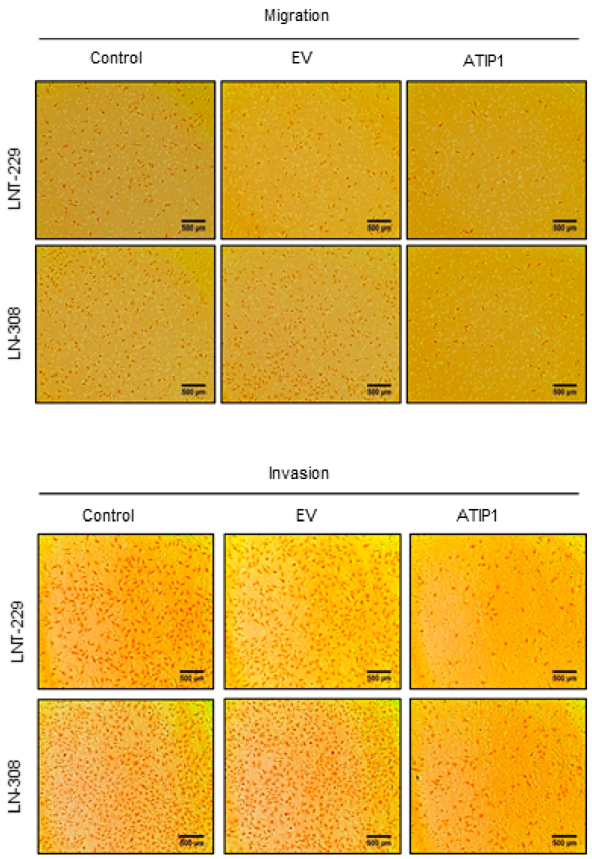

Figure S5. Elevated ATIP1 reduces migration and invasion. Parental (control), pcDNA3.1 (EV) or pcDNA3.1-ATIP1 (ATIP1) stably transfected LNT-229 and LN-308 cells were subjected for transwell migration and invasion assay. Transwell migration (upper panel) and invasion assays (lower panel) indicate a reduced migration and invasion capability of ATIP1 overexpressing glioma cells (scale = 500 μm).

Figure S6

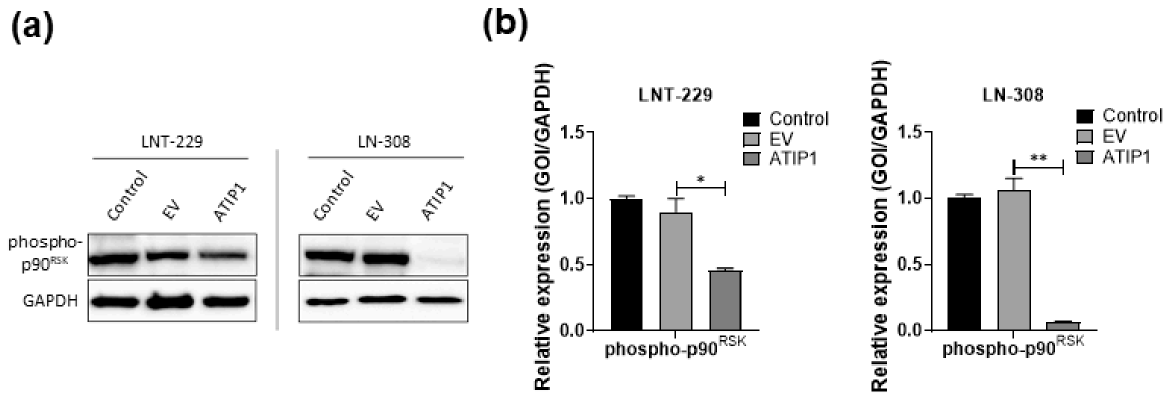

Figure S6. Phospho-p90^{RSK} expression is reduced in ATIP1 overexpressing glioma cells. a. Immunoblot of phospho-p90^{RSK} in parental (control), pcDNA3.1 (EV) and pcDNA3.1-ATIP1 (ATIP1) stably transfected LNT-229 and LN-308 cells. GAPDH served as the loading control. One representative blot is shown. b. Quantification of phospho-p90^{RSK} (n=3, SEM; * $P < 0.05$, ** $P < 0.01$).

Figure S7

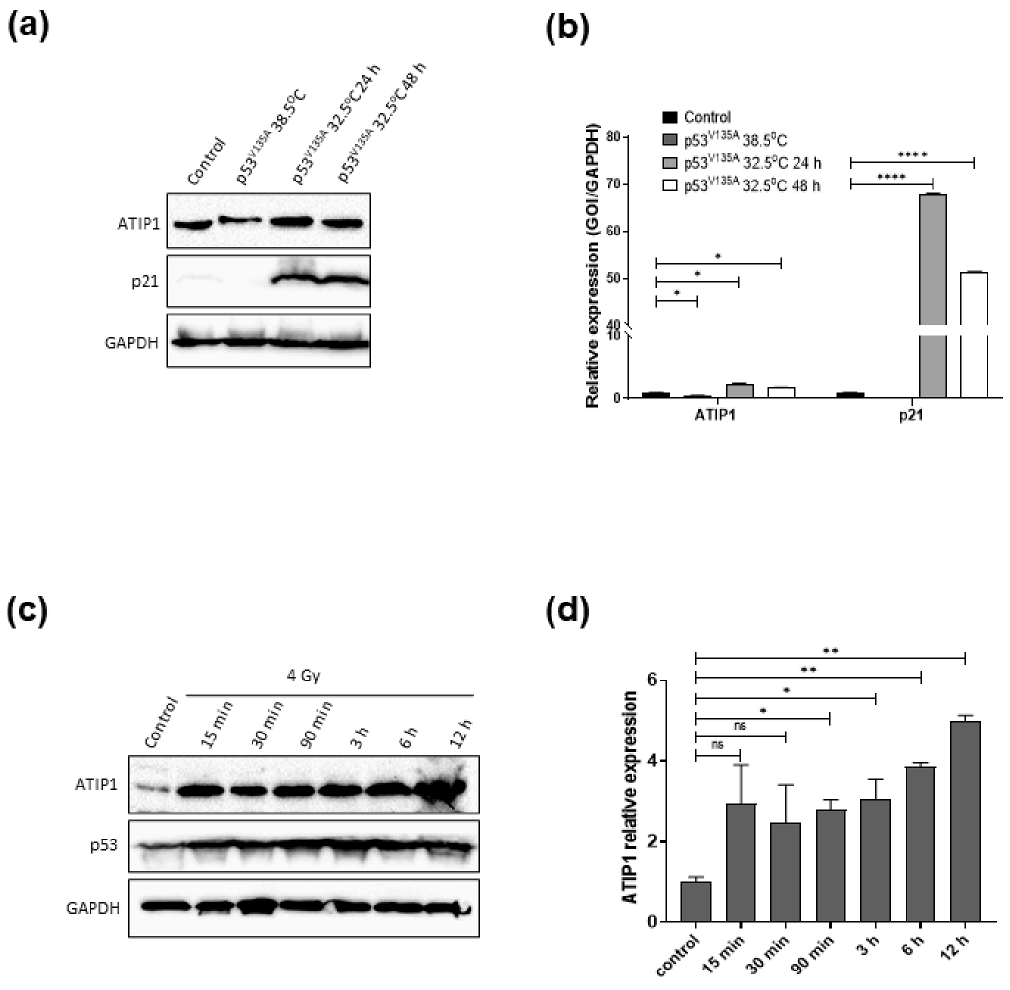

Figure S7. Irradiation induces ATIP1 expression. a. ATIP1 and p21 protein expression in parental (control) or $p53^{ts}$ expressing LNT-229 cells at the permissive (38.5°C) or $p53^{WT}$-inducing (32.5°C) temperature. p21 expression was assessed for the control of p53 activity. GAPDH serves as the loading control. b. Quantification of protein expression as indicated in a. (n=3, SEM; * $P < 0.05$, ** $P < 0.01$). c. Immunoblot of ATIP1 and p53 in LNT-229 cells irradiated with 4 Gy. GAPDH serves as a loading control (n=3, one representative blot is shown). d. Quantification of ATIP1 expression in control or irradiated LNT-229 cells as indicated in a. (n=3, SEM; ns: not significant, * $P < 0.05$, ** $P < 0.01$).

Figure S8

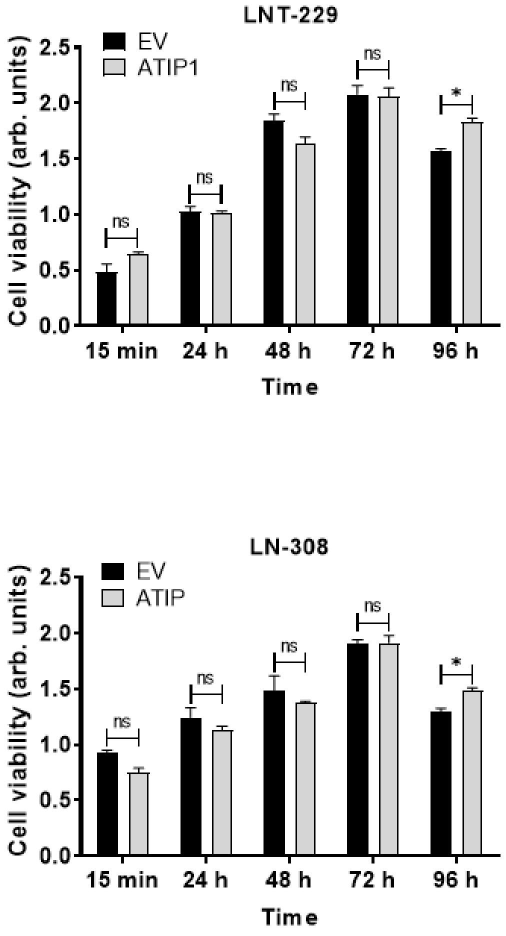

Figure S8. Elevated ATIP1 expression enhances the survival of irradiated glioma cells. Cell viability assay of pcDNA3.1 (EV) and pcDNA3.1-ATIP1 (ATIP1) stably transfected LNT-229 and LN-308 cells. The cells were irradiated with 4 Gy and cell viability was measured using the MTT assay at the indicated time points after irradiation (n=3, SEM; ns: not significant, * $P < 0.05$).

Figure S9

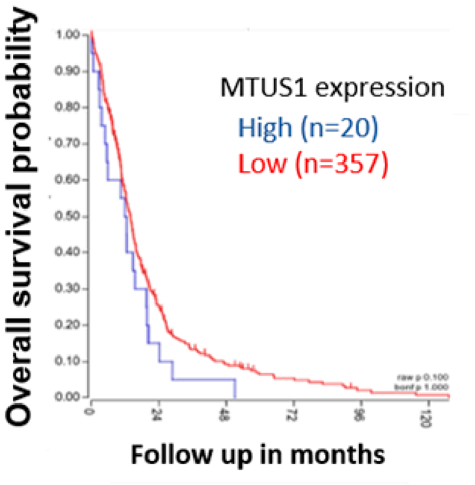

Figure S9. Correlation of ATIP1 expression with the survival of glioma patients receiving irradiation therapy (R2 database mining). Overall survival probability of glioma patients that received tumor irradiation. Patients with high *MTUS1* expression (n=20) showed worse overall survival compared to patients showing low *MTUS1* expression in the tumor (n=357).

Figure S10

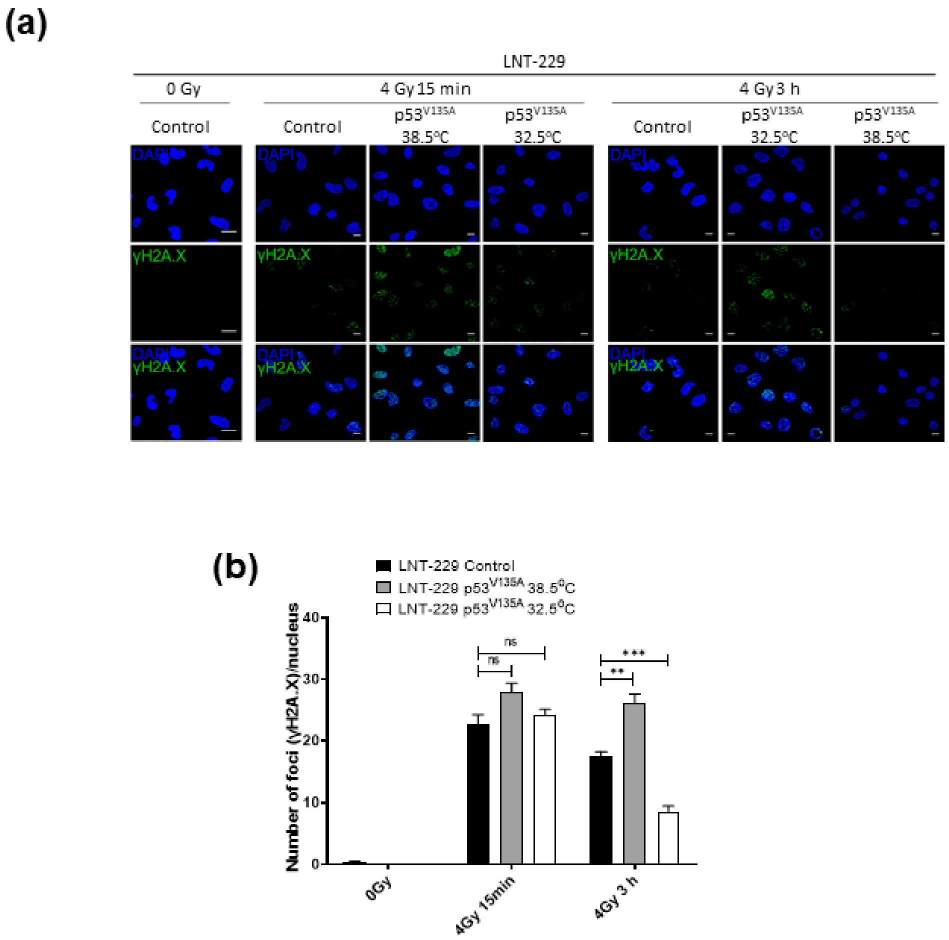

Figure S10. p53 expression modulates DNA repair dynamics. a. γH2A.X foci in parental (control) or $p53^{ts}$ expressing LNT-229 cells at the p53 permissive (38.5°C) or $p53^{WT}$-inducing (32.5°C) temperature. b. Quantification of γH2A.X foci/cell as indicated in b. (n=4, SEM; ns: not significant, ** $P < 0.01$, *** $P < 0.001$).

References

1. Happold, C.; Roth, P.; Wick, W.; Schmidt, N.; Florea, A.M.; Silginer, M.; Reifenberger, G.; Weller, M. Distinct molecular mechanisms of acquired resistance to temozolomide in glioblastoma cells. *J Neurochem* **2012**, *122*, 444-455, doi:10.1111/j.1471-4159.2012.07781.x.
2. Mantwill, K.; Naumann, U.; Seznec, J.; Girbinger, V.; Lage, H.; Surowiak, P.; Beier, D.; Mittelbronn, M.; Schlegel, J.; Holm, P.S. YB-1 dependent oncolytic adenovirus efficiently inhibits tumor growth of glioma cancer stem like cells. *Journal of Translational Medicine* **2013**, *11*, 1-13.
3. Beier, C.P.; Rasmussen, T.; Dahlrot, R.H.; Tenstad, H.B.; Aaro, J.S.; Sorensen, M.F.; Heimisdottir, S.B.; Sorensen, M.D.; Svenningsen, P.; Riemenschneider, M.J., et al. Aberrant neuronal differentiation is common in glioma but is associated neither with epileptic seizures nor with better survival. *Sci Rep* **2018**, *8*, 14965, doi:10.1038/s41598-018-33282-5.
4. Ishii, N.; Maier, D.; Merlo, A.; Tada, M.; Sawamura, Y.; Diserens, A.-C.; Meir, E.G.V. Frequent Co-Alterations of TP53, p16/CDKN2A, p14ARF, PTEN Tumor Suppressor Genes in Human Glioma Cell Lines. *Brain Pathology* **1999**, *9*, 469-479.
5. Hermisson, M.; Klumpp, A.; Wick, W.; Wischhusen, J.; Nagel, G.; Roos, W.; Kaina, B.; Weller, M. O6-methylguanine DNA methyltransferase and p53 status predict temozolomide sensitivity in human malignant glioma cells. *J Neurochem* **2006**, *96*, 766-776, doi:10.1111/j.1471-4159.2005.03583.x.

Uncropped original western blots as presented in the article figures

Fig. 1(b)

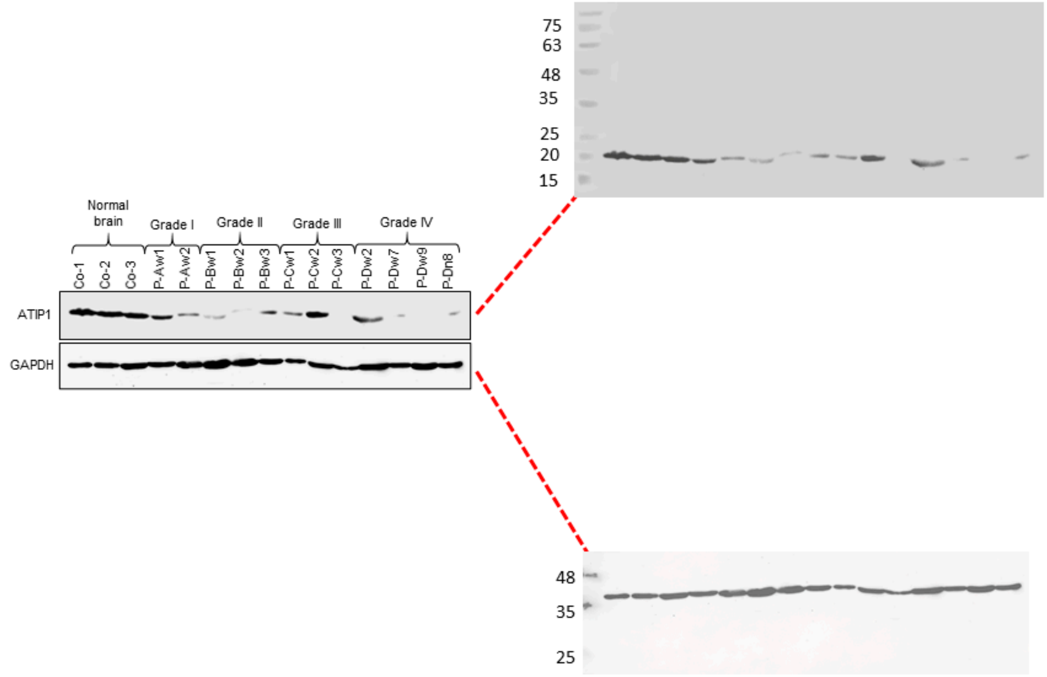

Fig. 1(i)

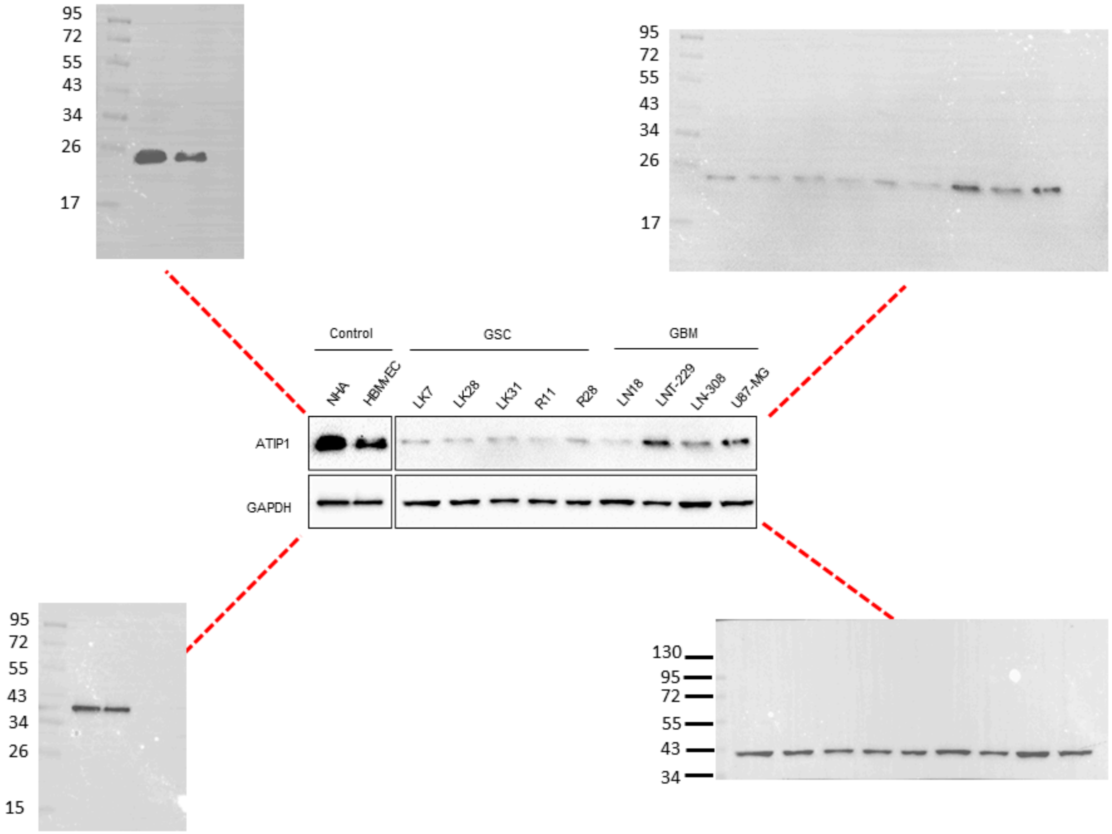

Fig. 2(c)

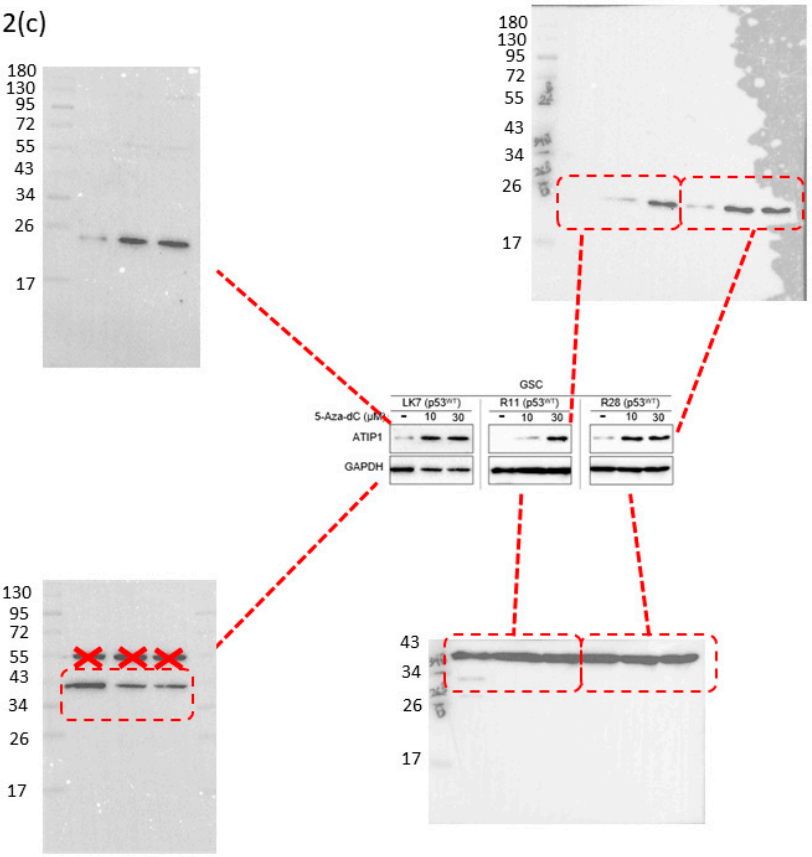
GSC
LK7 (p53WT)
R11 (p53WT)
R28 (p53WT)
5-Aza-dC (μM)
- 10 30
ATIP1
GAPDH
180
130
95
72
55
43
34
26
17

Fig. 2(c)

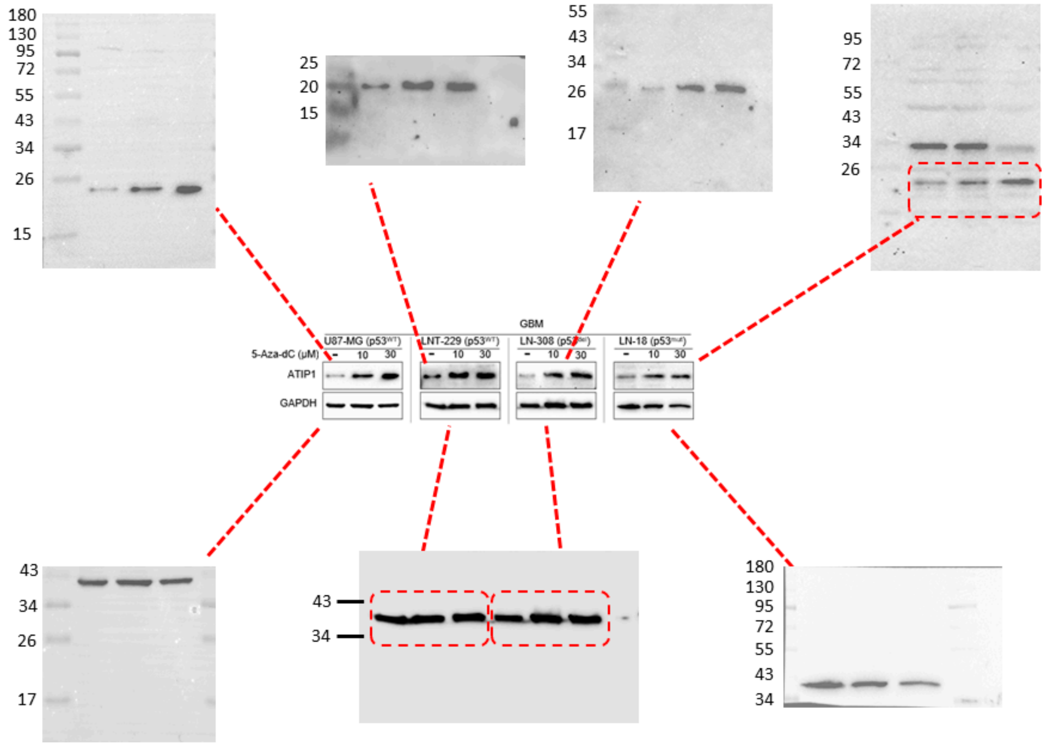
GBM
U87-MG (p53WT)
LNT-229 (p53WT)
LN-308 (p53Del)
LN-18 (p53mut)
5-Aza-dC (μM)
- 10 30
ATIP1
GAPDH
180
130
95
72
55
43
34
26
25
20
15
17

Fig. 3(d)

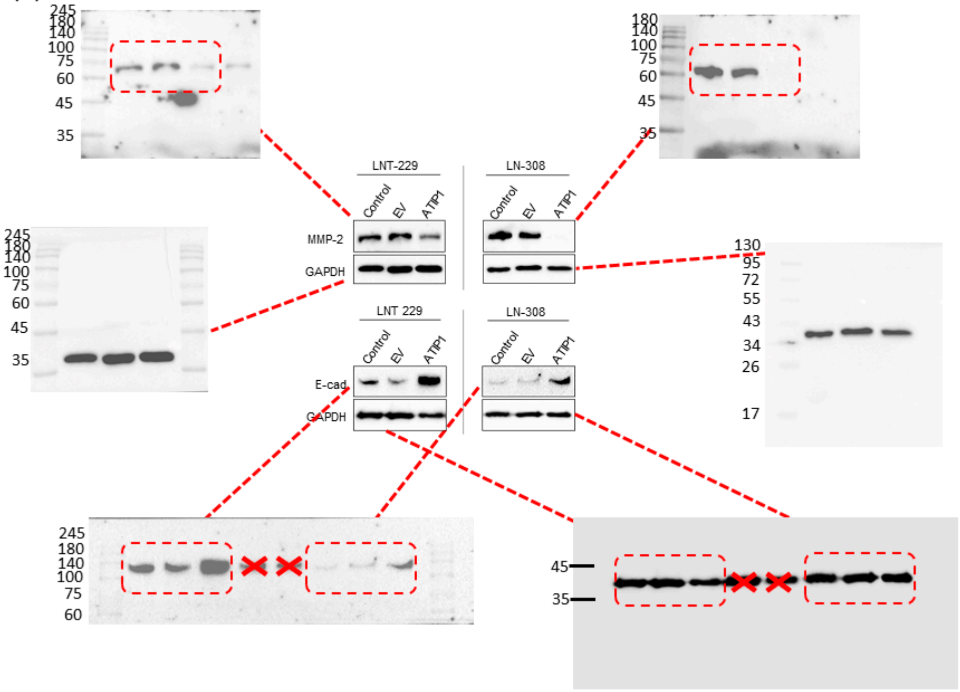
LNT-229
LN-308
Control
EV
ATIP1
MMP-2
GAPDH
LNT 229
E-cad
245
180
140
100
75
60
45
35
130
95
72
55
43
34
26
17

Fig. 4(a)

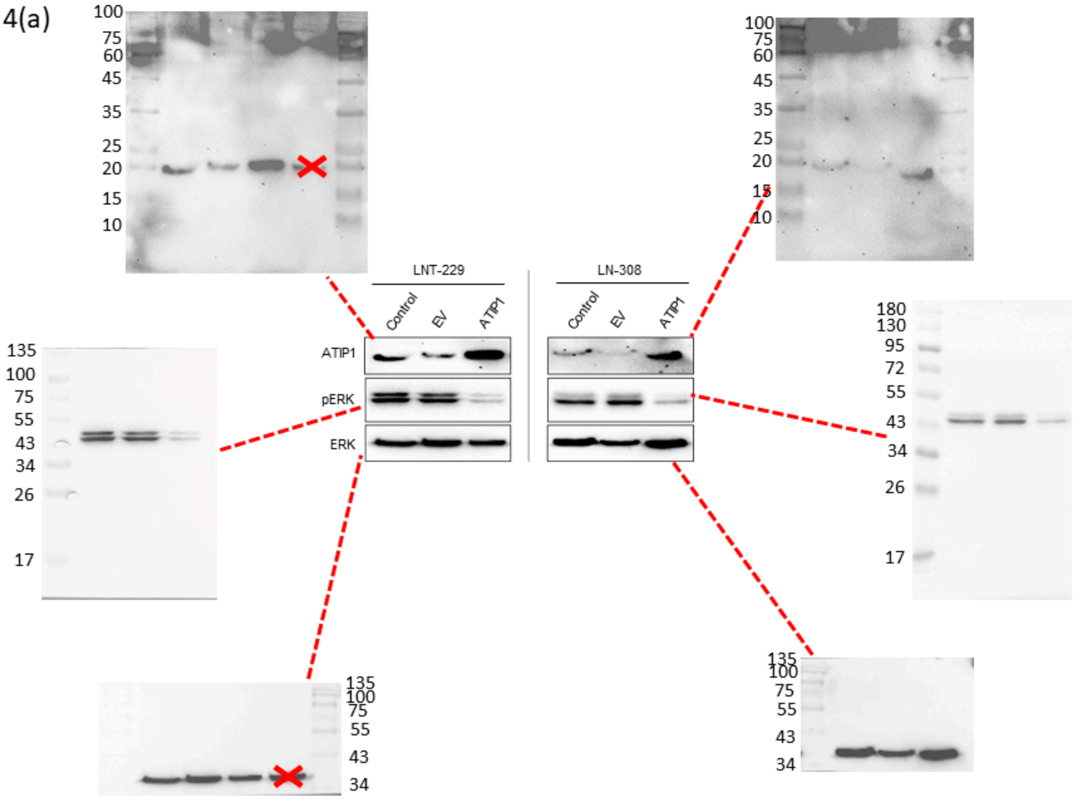
LNT-229
LN-308
Control
EV
ATIP1
pERK
ERK
100
75
60
45
35
25
20
15
10
135
55
43
34
26
17
180
130
95
72

Fig. 4(a)

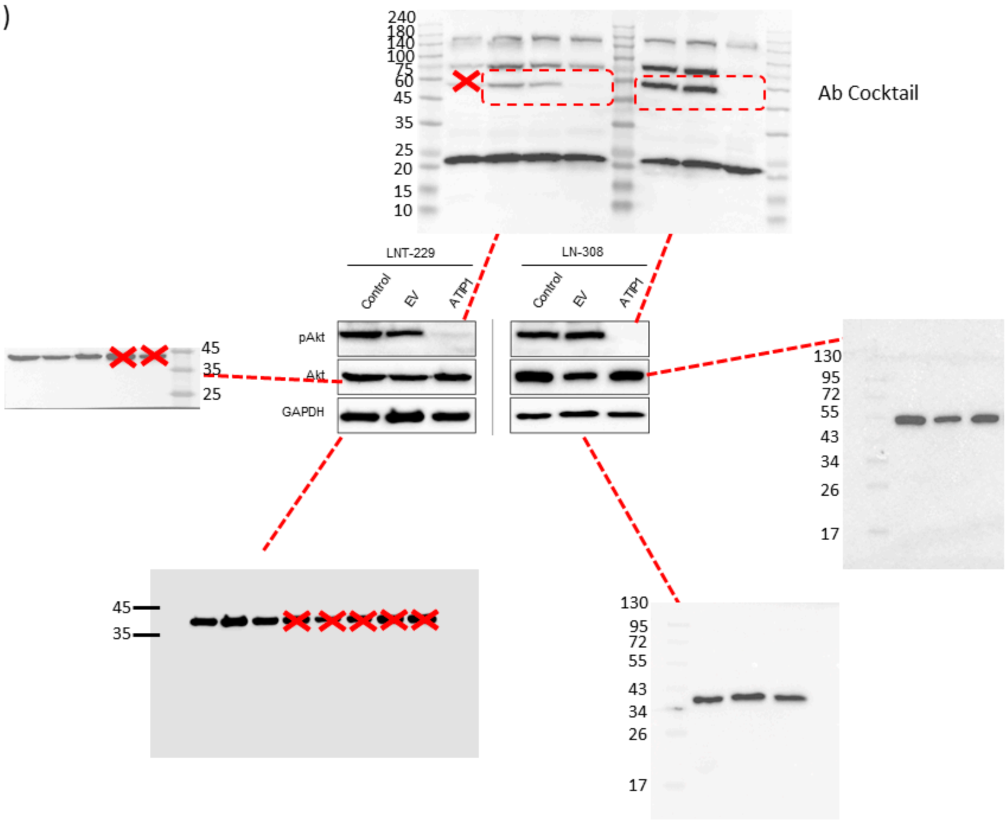
Ab Cocktail
LNT-229
LN-308
Control
EV
ATIP1
pAkt
Akt
GAPDH

Fig. 4(a)

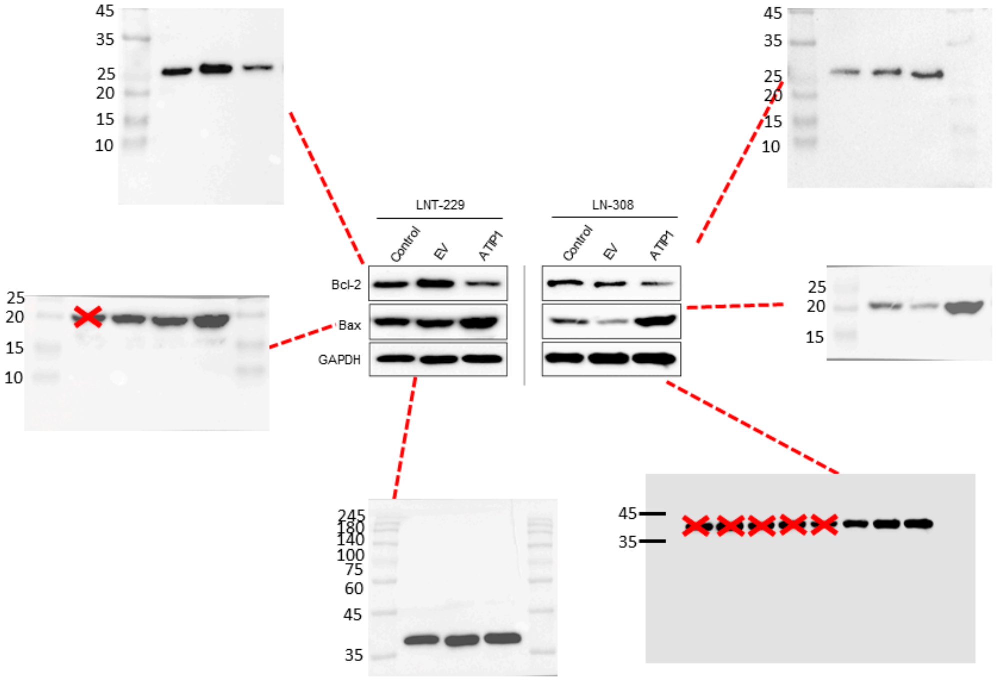
LNT-229
LN-308
Control
EV
ATIP1
Bcl-2
Bax
GAPDH

Fig. 4(c)

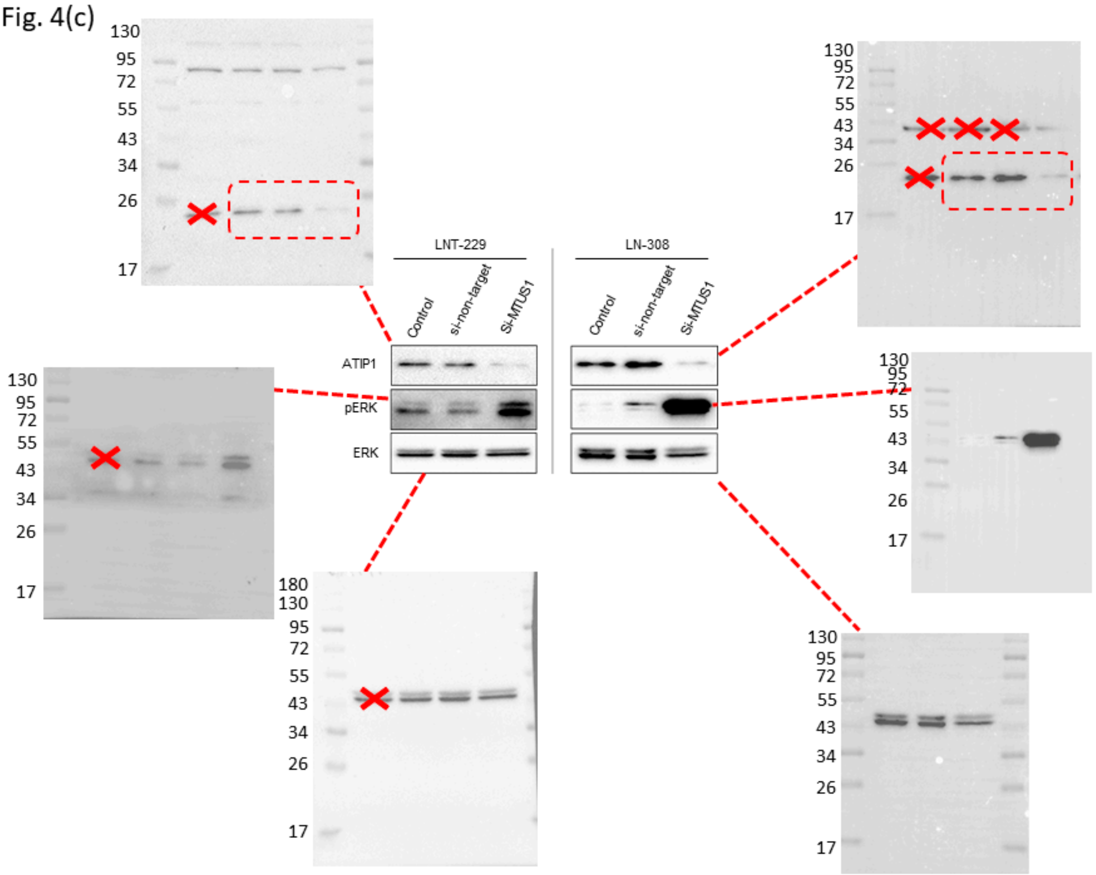
LNT-229
LN-308
Control
si-non-target
Si-MTUS1
ATIP1
pERK
ERK
130
95
72
55
43
34
26
17
180

Fig. 4(c)

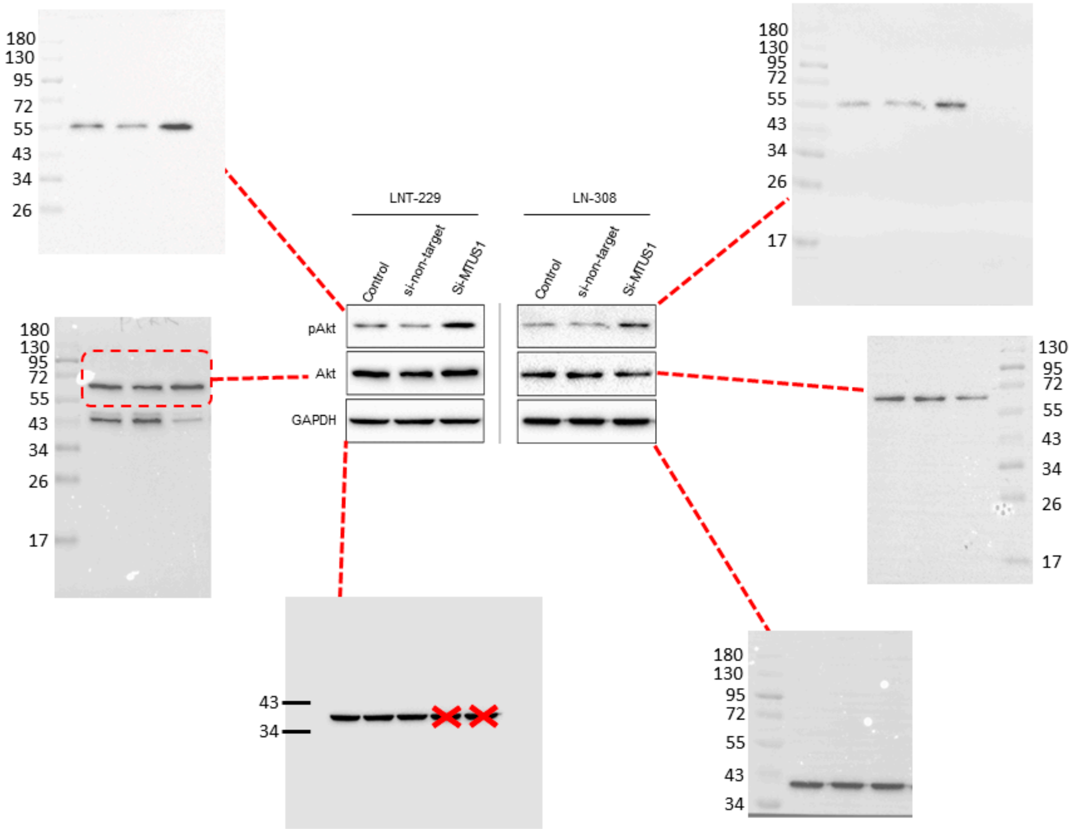
LNT-229
LN-308
Control
si-non-target
Si-MTUS1
pAkt
Akt
GAPDH
180
130
95
72
55
43
34
26
17

Fig. 4(c)

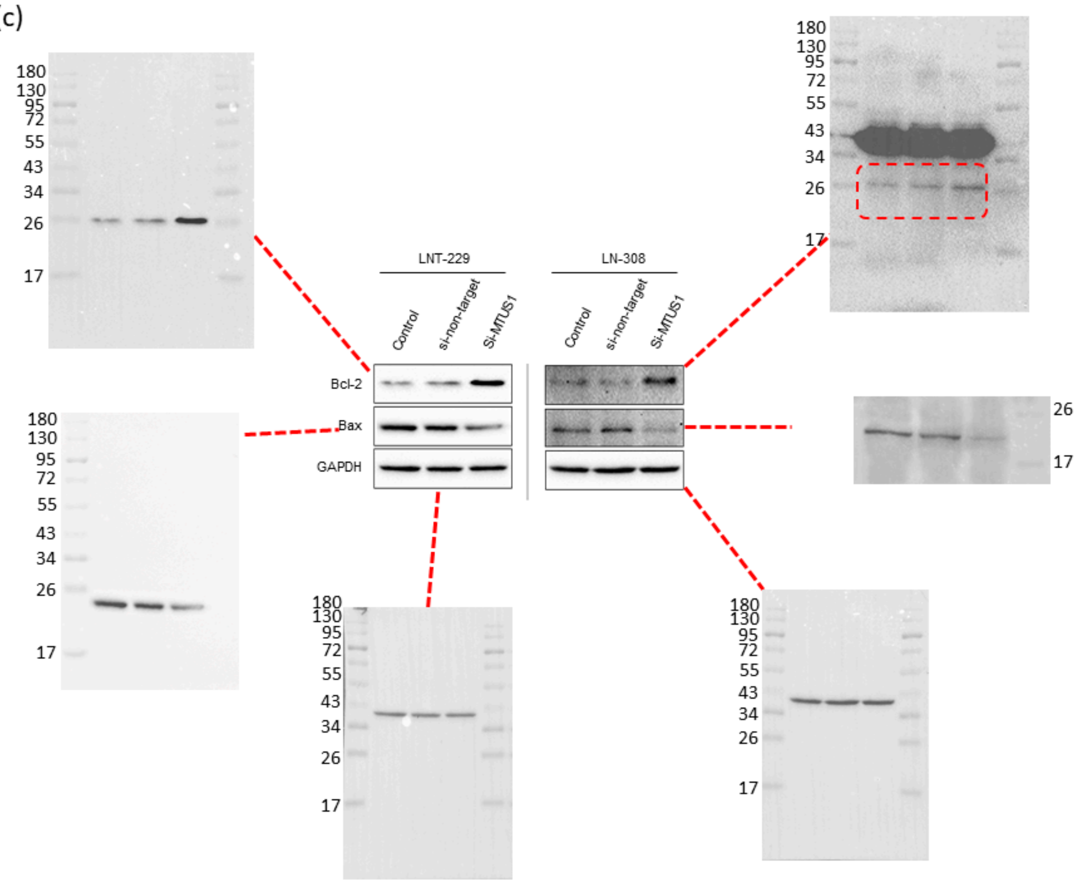
LNT-229
LN-308
Control
si-non-target
Si-MTUS1
Bcl-2
Bax
GAPDH
180
130
95
72
55
43
34
26
17

Fig. 4(e)

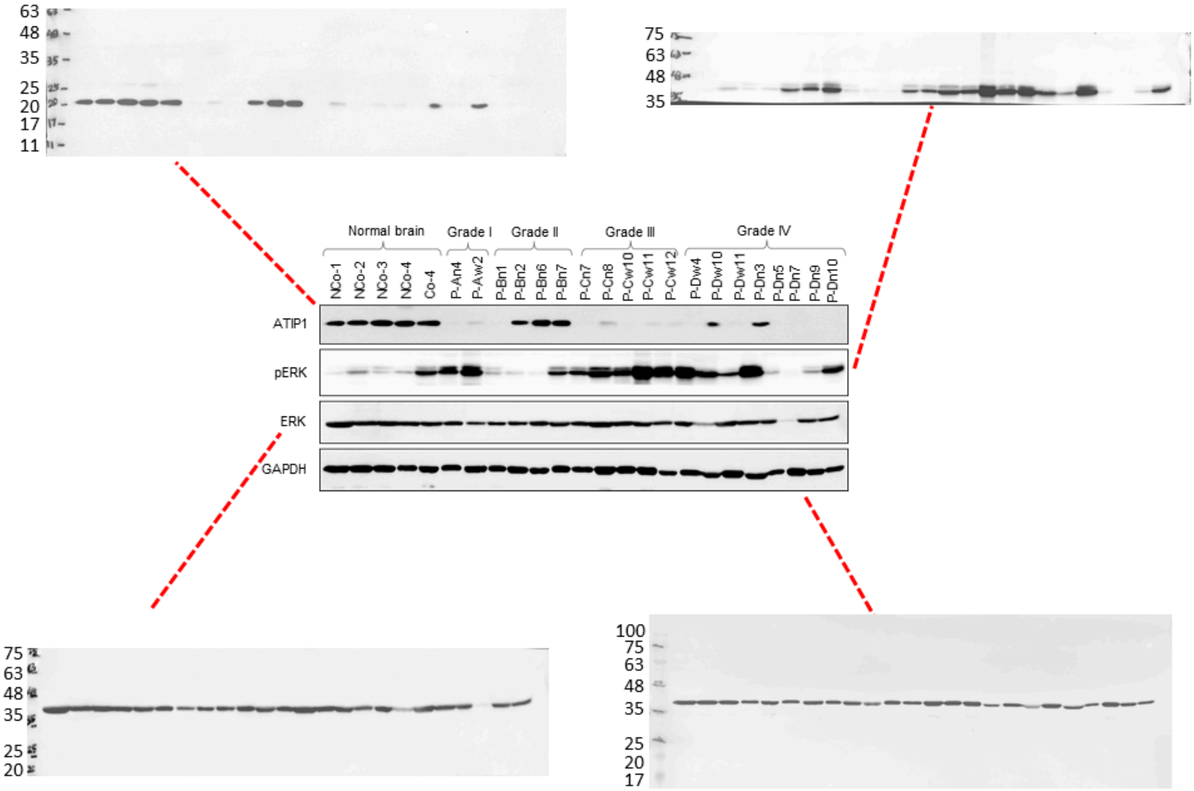
Normal brain
Grade I
Grade II
Grade III
Grade IV
NCo-1
NCo-2
NCo-3
NCo-4
Co-4
P-An4
P-Aw2
P-Bn1
P-Bn2
P-Bn6
P-Bn7
P-Cn7
P-Cn8
P-Cw10
P-Cw11
P-Cw12
P-Dw4
P-Dw10
P-Dw11
P-Dn3
P-Dn5
P-Dn7
P-Dn9
P-Dn10
ATIP1
pERK
ERK
GAPDH
100
75
63
48
35
25
20
17
11

Fig. 5(a)

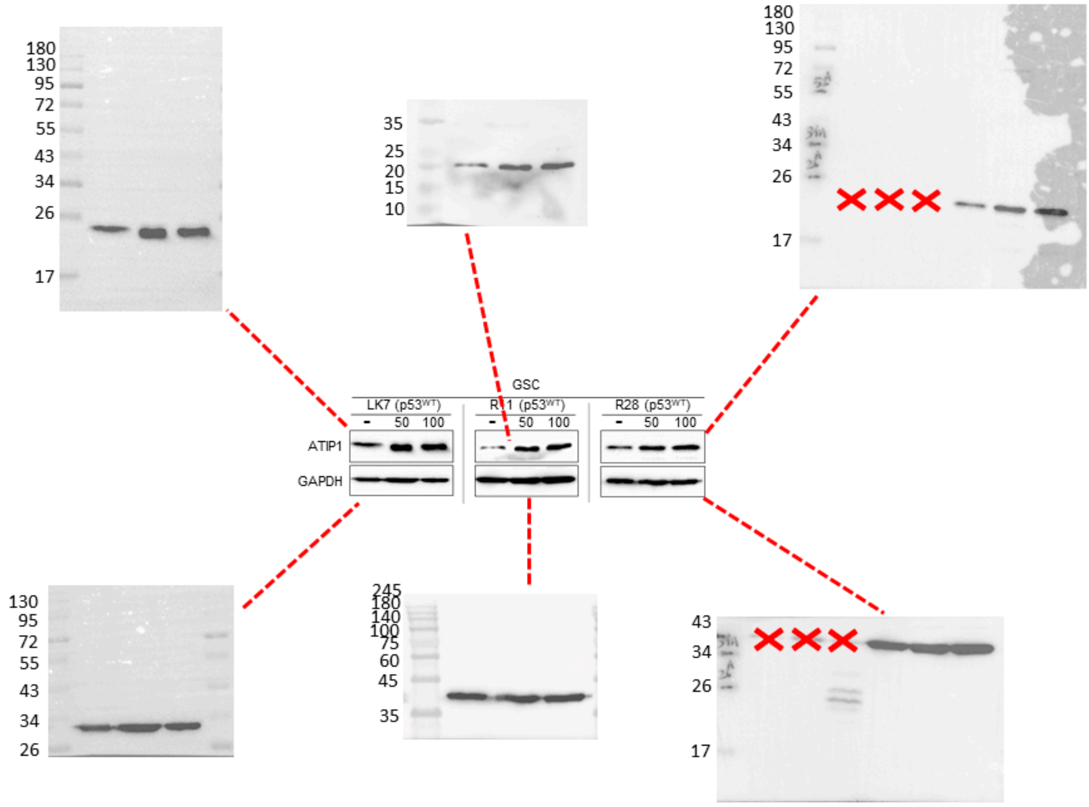
GSC
LK7 (p53WT)
R11 (p53WT)
R28 (p53WT)
- 50 100
ATIP1
GAPDH

Fig. 5(a)

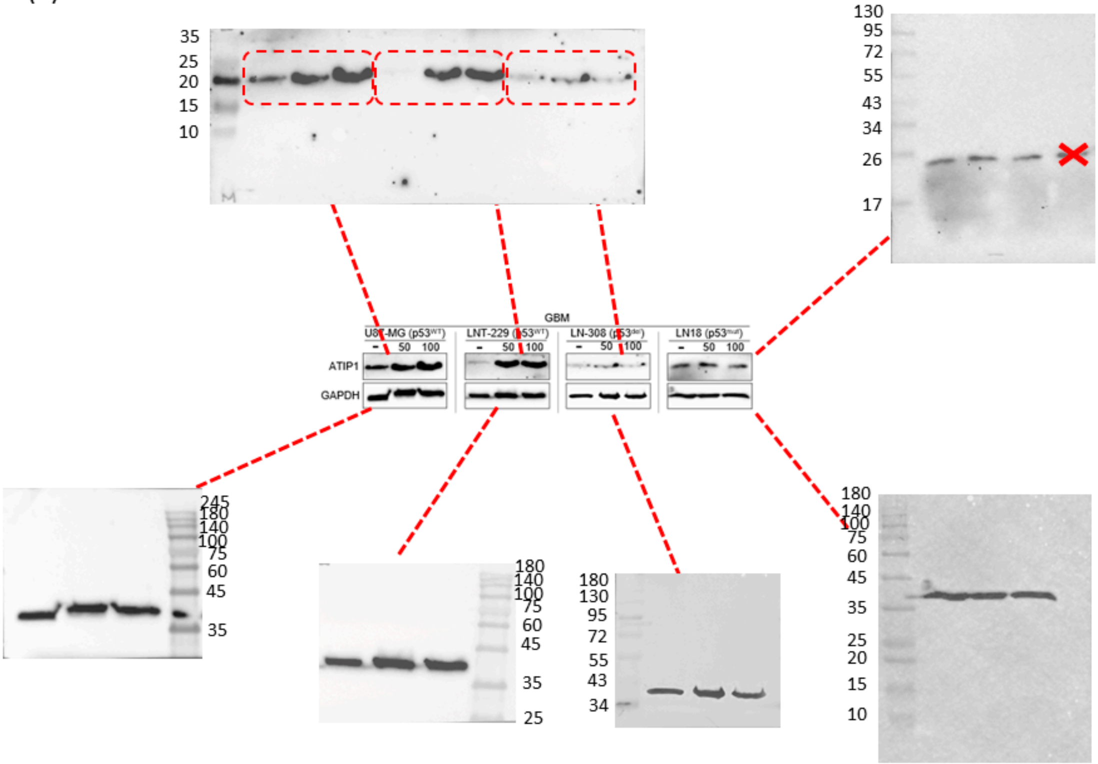
GBM
U87-MG (p53WT)
LNT-229 (p53WT)
LN-308 (p53
LN18 (p53
- 50 100
ATIP1
GAPDH

Fig. 5(e)

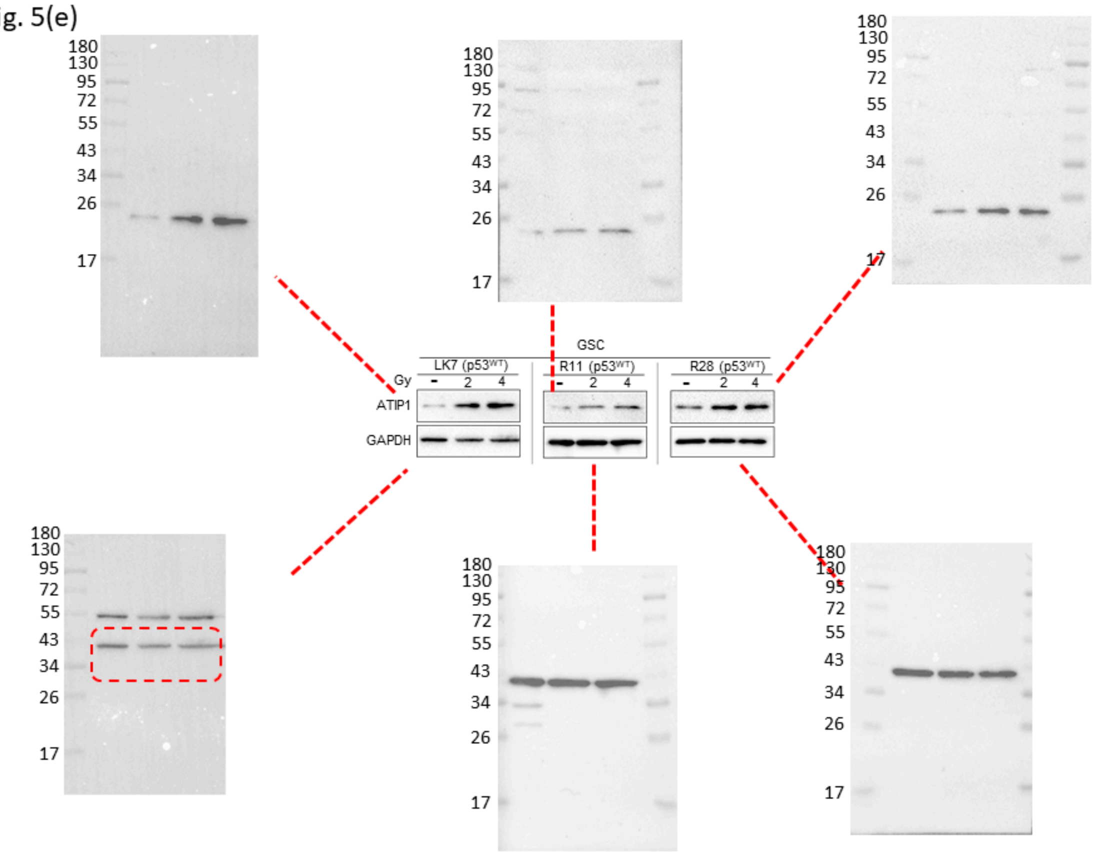
GSC
LK7 (p53WT)
R11 (p53WT)
R28 (p53WT)
Gy
-
2
4
ATIP1
GAPDH
180
130
95
72
55
43
34
26
17

Fig. 5(e)

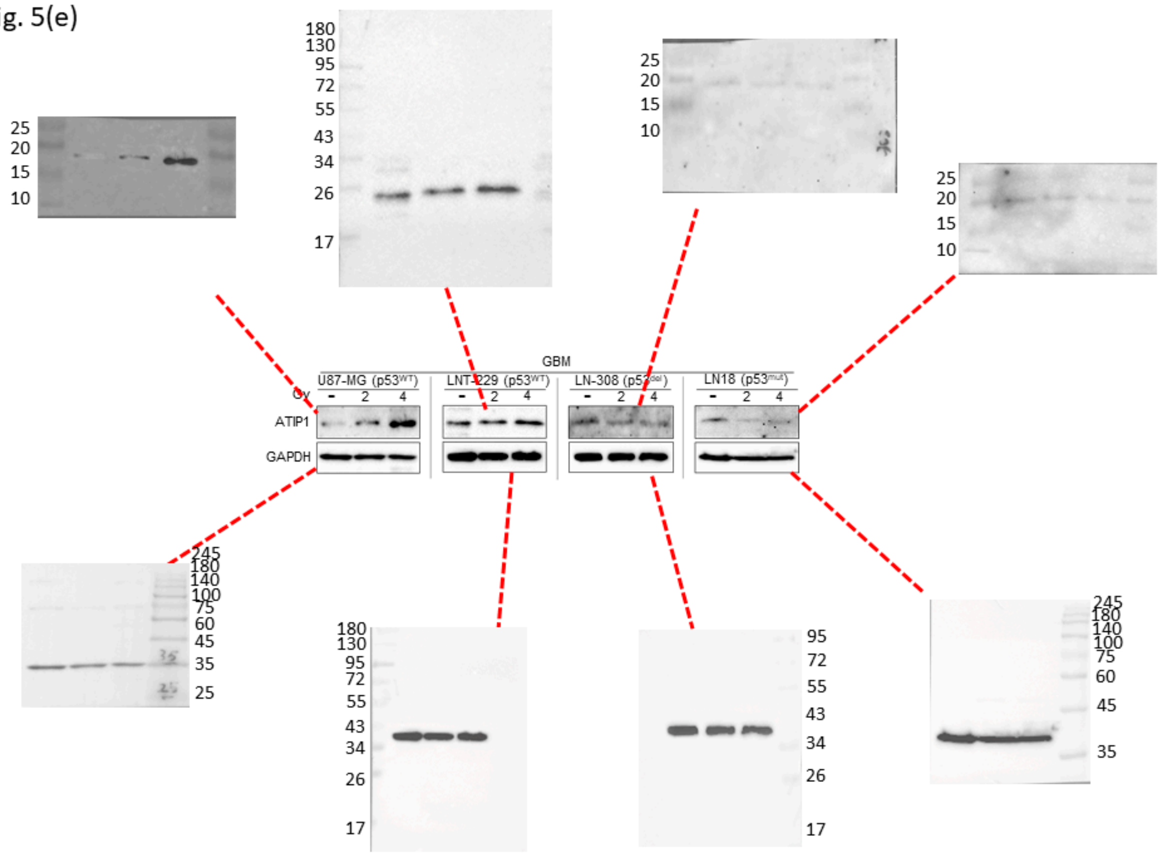
GBM
U87-MG (p53WT)
LNT-229 (p53WT)
LN-308 (p53del)
LN18 (p53mut)
Gy
-
2
4
ATIP1
GAPDH
25
20
15
10
180
130
95
72
55
43
34
26
17
245
140
100
75
60
45
35

Figure S1(a)

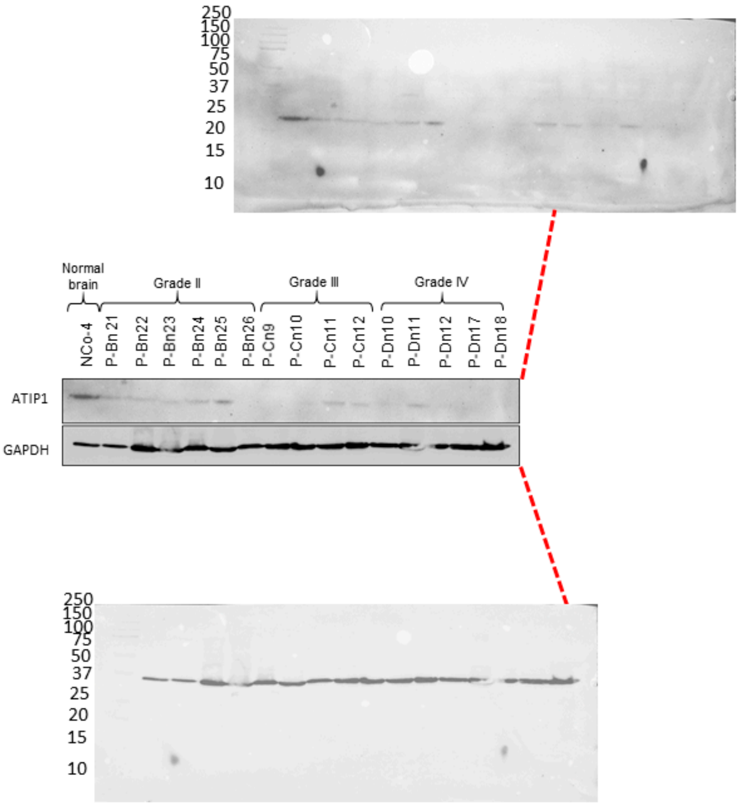

Figure S2(a)

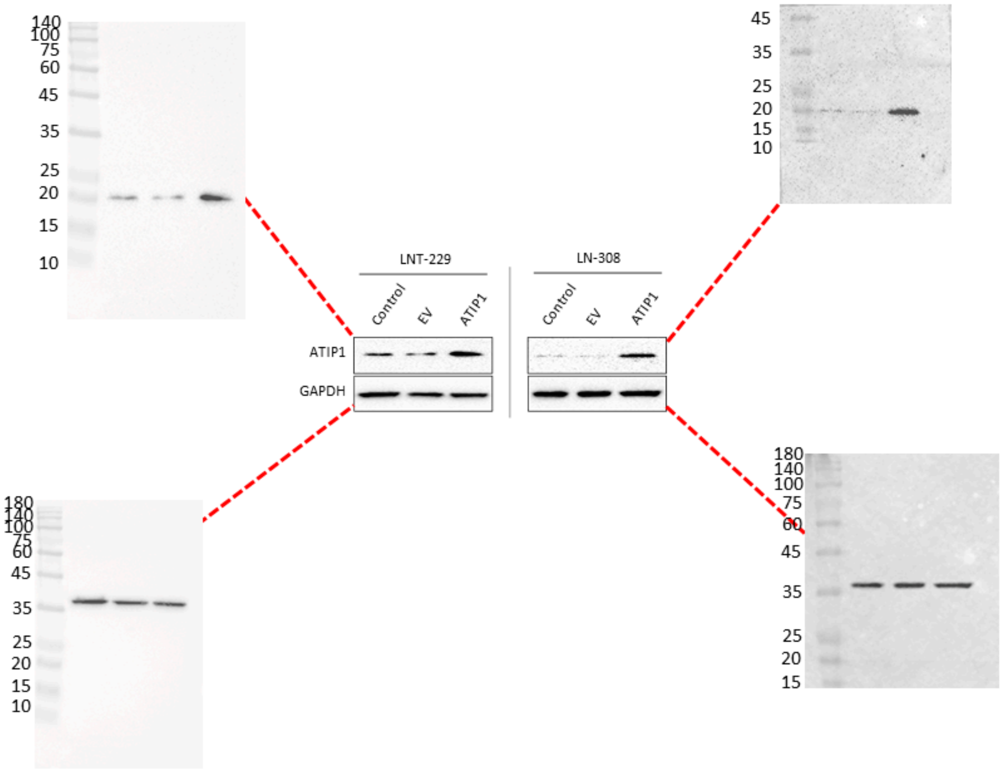

Figure S6(a)

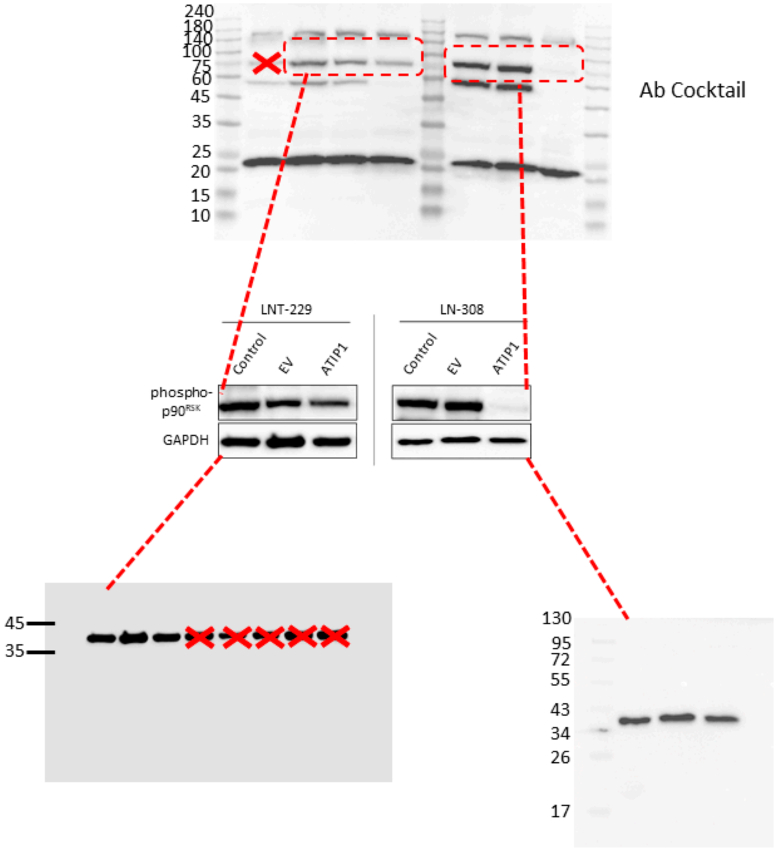

Figure S7(a)

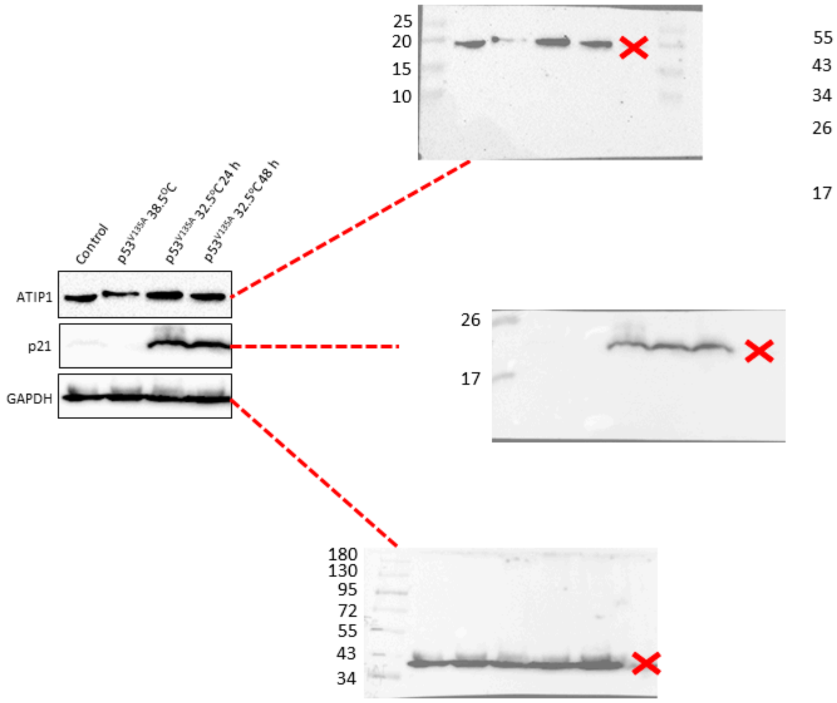

Figure S7(c)

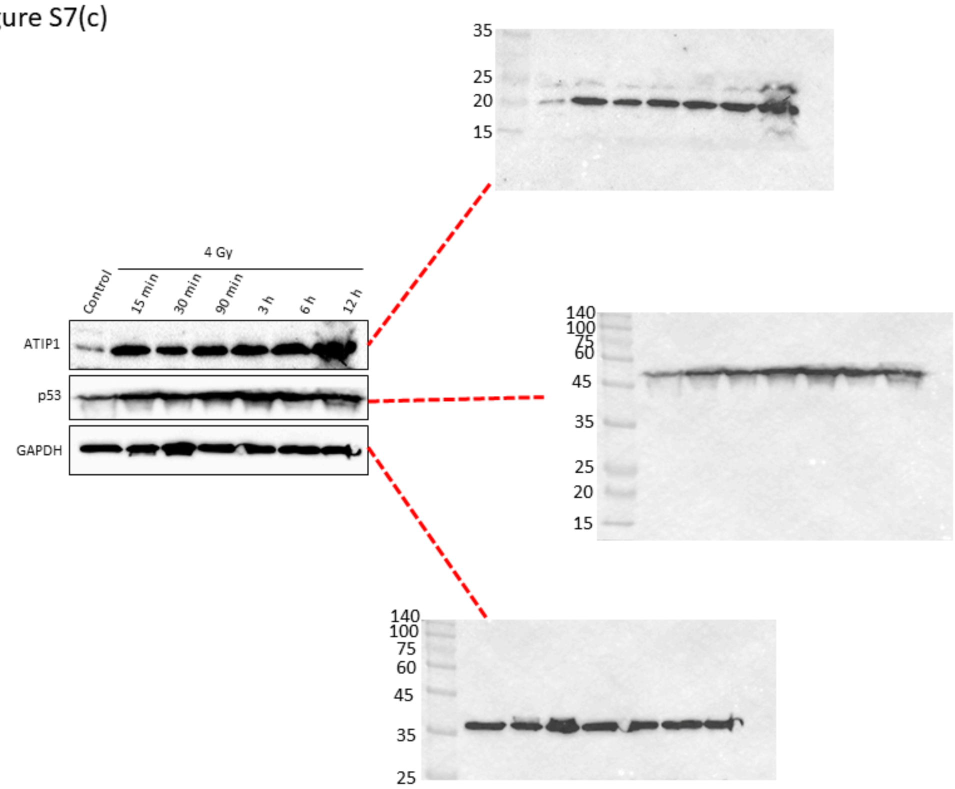
4 Gy
Control
15 min
30 min
90 min
3 h
6 h
12 h
ATIP1
p53
GAPDH
35
25
20
15
140
100
75
60
45
35
25
20
15
140
100
75
60
45
35
25
